# Insights Into the Species-Specific Microbiota of Greenideinae (Hemiptera: Aphididae) With Evidence of Phylosymbiosis

**DOI:** 10.3389/fmicb.2022.828170

**Published:** 2022-02-22

**Authors:** Man Qin, Jing Chen, Liyun Jiang, Gexia Qiao

**Affiliations:** ^1^Key Laboratory of Zoological Systematics and Evolution, Institute of Zoology, Chinese Academy of Sciences, Beijing, China; ^2^College of Life Sciences, University of Chinese Academy of Sciences, Beijing, China

**Keywords:** phylosymbiosis signal, microbiota variation, aphid species-specific, host plant, symbiont diversity

## Abstract

Aphids and their symbionts represent an outstanding model for studies of insect–symbiont interactions. The aphid microbiota can be shaped by aphid species, geography and host plants. However, the relative importance of phylogenetic and ecological factors in shaping microbial community structures is not well understood. Using Illumina sequencing of the V3–V4 hypervariable region of the 16S rRNA gene, we characterized the microbial compositions of 215 aphid colonies representing 53 species of the aphid subfamily Greenideinae from different regions and plants in China, Nepal, and Vietnam. The primary endosymbiont *Buchnera aphidicola* and secondary symbiont *Serratia symbiotica* dominated the microbiota of Greenideinae. We simultaneously explored the relative contribution of host identity (i.e., aphid genus and aphid species), geography and host plant to the structures of bacterial, symbiont and secondary symbiont communities. Ordination analyses and statistical tests highlighted the strongest impact of aphid species on the microbial flora in Greenideinae. Furthermore, we found a phylosymbiosis pattern in natural Greenideinae populations, in which the aphid phylogeny was positively correlated with microbial community dissimilarities. These findings will advance our knowledge of host-associated microbiota assembly across both host phylogenetic and ecological contexts.

## Introduction

Sap-feeding aphids harbor a diverse community of microbes, predominantly symbionts (Jousselin et al., 2016; Guyomar et al., 2018), which represent an excellent model to study symbiosis in insects. Almost all aphids possess the primary endosymbiont *Buchnera aphidicola* within specialized bacteriocytes to compensate for the nutritional deficiency in their diet (Baumann et al., 1995). *Buchnera* is inherited by strict vertical transmission (Koga et al., 2012) and cospeciates with aphids (Munson et al., 1991; Clark et al., 2000; Xu et al., 2018). In addition, aphids host several types of secondary symbionts that are not essential for survival. The beneficial roles of secondary symbionts have been demonstrated in numerous studies, including protection against parasitoid wasps and fungi (Oliver et al., 2003; Scarborough et al., 2005; Heyworth and Ferrari, 2015), tolerance to heat shock (Chen et al., 2000; Montllor et al., 2002; Guay et al., 2009) and changes in host plant range (Tsuchida et al., 2004; Wagner et al., 2015). Secondary symbionts usually reside in secondary bacteriocytes, sheath cells, or the hemocoel (Fukatsu et al., 2000) and undergo maternal transmission and occasionally horizontal transmission (Russell et al., 2003; Michalik et al., 2014; Pons et al., 2019). To date, nine secondary symbionts have been most frequently studied in aphids, namely, *Arsenophonus* (Russell et al., 2003), *Fukatsuia symbiotica* (Guay et al., 2009), *Hamiltonella defensa* (Darby et al., 2001), *Regiella insecticola* (Sandström et al., 2001), *Rickettsia* (Chen et al., 1996), *Rickettsiella viridis* (Tsuchida et al., 2010), *Serratia symbiotica* (Unterman et al., 1989), *Spiroplasma* (Fukatsu et al., 2001), and *Wolbachia* (Augustinos et al., 2011).

Numerous studies have investigated the ecological factors shaping aphid-symbiotic community structures and highlighted the importance of geography (Tsuchida et al., 2002; Sepúlveda et al., 2017; Gallo-Franco et al., 2019) and host plants (Simon et al., 2003; Ferrari et al., 2012; Brady and White, 2013; Xu S. F. et al., 2020). Symbiont infection patterns were also related to aphid species (Qin et al., 2021a), characteristics of aphids (Xu et al., 2021), and seasonal shifts (Smith et al., 2015; Liu et al., 2019). Considering the impact of host phylogeny, some studies substantiated the correlation between microbiota dissimilarities and aphid relatedness (McLean et al., 2019; Qin et al., 2021a,b), whereas the phylogenetic signature was not detected in some aphid groups (e.g., Eriosomatinae and Hormaphidinae) (Xu T. T. et al., 2020; Xu et al., 2021).

Phylosymbiosis is a term used to describe the eco-evolutionary pattern, in which the microbial community compositional similarity is significantly correlated with host phylogeny (Brucker and Bordenstein, 2013; Lim and Bordenstein, 2020). This pattern does not assume stable and long-term associations between hosts and microbes. Both host-microbe codiversification and ecological filtering by conserved host traits can contribute to the mechanisms underpinning phylosymbiosis (Sanders et al., 2014; Mazel et al., 2018; Lim and Bordenstein, 2020). Evidence for phylosymbiosis has been provided in insects, birds and mammals (Brooks et al., 2016; Groussin et al., 2017; Chiarello et al., 2018; Hammer et al., 2020; Trevelline et al., 2020). Phylosymbiosis signals are variable across host clades and may be weakened or even erased over extended evolutionary periods (Mazel et al., 2018). However, the extent to which host phylogenetic scales (e.g., from intraspecific to family levels) play a role in phylosymbiosis detection remains to be explored.

Greenideinae is a subfamily of Aphididae within the Hemiptera order, in which 179 extant species and 10 fossil species belonging to 18 genera have been recognized worldwide (Favret, 2021). Greenideinae comprises three tribes, namely, Cervaphidini, Greenideini, and Schoutedeniini. Cervaphidini contains one fossil genus and six extant genera including *Anomalosiphum* and *Cervaphis*. Greenideini is represented by *Allotrichosiphum*, *Eutrichosiphum*, *Greenidea*, *Greenideoida*, *Mesotrichosiphum*, *Mollitrichosiphum*, and *Tritrichosiphum*. Schoutedeniini consists of *Eonaphis*, *Paulianaphis*, *Schoutedenia*, and *Palaeogreenidea* (fossil genus). Greenideinae is monoecious, with a holocyclic or anholocyclic life cycle. Host plants of Schoutedeniini species belong primarily to Euphorbiaceae (Blackman and Eastop, 2021). Cervaphidini colonizes plants from Fagaceae, Fabaceae, Myrtaceae, Tiliaceae, and so on. The majority of Greenideini species feed on young leaves or shoots of Fagaceae. Other plants have also been reported to serve as hosts of Greenideini, such as Betulaceae, Juglandaceae, and Moraceae. Some Greenideini species exhibit high host plant diversity and colonize plants from different families, whereas many species feed on few types of plants. Greenideinae is mainly distributed in eastern and southern Asia, with 92 species recorded in China (Blackman and Eastop, 2021). Some Greenideinae species, such as *Greenidea ficicola* and *Greenidea psidii*, are insect pests that seriously threaten the economics of agriculture and horticulture. Qin et al. (2021a) uncovered the phylosymbiotic signatures of the microbial community associated with *Mollitrichosiphum* at the genus level and revealed intraspecific phylosymbiosis within one aphid species, *M. tenicroperus* (Qin et al., 2021b). However, little is known about the microbiota structures across the aphid subfamily Greenideinae. Greenideinae provides an opportunity to explore whether phylosymbiotic patterns occur at various host taxonomic levels in a specific aphid group.

Based on high-throughput 16S rRNA gene sequencing, we aimed to reveal the microbial community composition associated with Greenideinae. We fully assessed the contribution of host identity (i.e., aphid genus and aphid species), geographic distribution and host plant on bacterial, symbiont (including *Buchnera* and secondary symbionts) and secondary symbiont communities. To further understand the ecological and evolutionary processes of microbiota assemblages, we explored the presence of a phylosymbiotic pattern in Greenideinae.

## Materials and Methods

### Aphid Collection and DNA Extraction

We sampled 215 aphid colonies from 29 plant families and 32 geographic regions of China, Nepal, and Vietnam, representing 53 species and 9 genera within Greenideinae (Table 1). Information regarding sample collection is provided in Supplementary Table 1. The samples for slide mounting were preserved in 75% ethanol, and the specimens for molecular experiments were stored in 95% ethanol at −20°C. All the samples were deposited in the National Animal Collection Resource Center, Institute of Zoology, Chinese Academy of Sciences, Beijing, China. Aphid identification was performed using morphological examination and DNA barcoding.

**TABLE 1:** Sampling information for the Greenideinae samples used in the present study.

Aphid tribe	Aphid genus	Aphid species	Number of samples
Cervaphidini	*Anomalosiphum*	*A. takahashii*, *A. tiomanensis*	4
	*Cervaphis*	*C. quercus*, *C. rappardi*	5
Greenideini	*Allotrichosiphum*	*A. cyclobalanopsidis*	2
	*Eutrichosiphum*	*E. alnicoia*, *E. alnifoliae*, *E. apicifuscum*, *E. dubium*, *E. heterotrichum*,	49
		*E. khasyanum*, *E. kumaoni*, *E. parvulum*, *E. pasaniae*, *E. pseudopasaniae*,	
		*E. sinense*, *E.* sp. 1, *E.* sp. 2, *E. tattakanum*	
	*Greenidea*	*G. anonae*, *G. ayyari*, *G. brideliae*, *G. bucktonis*, *G. camelliae*,	76
		*G. castanopsidis G. cayratiae*, *G. decaspermi*, *G. ficicola*, *G. flacourtiae*,	
		*G. kuwanai*, *G. nigra*, *G. nipponica*, *G. prunicola*, *G. psidii*,	
		*G. querciphaga*, *G.* sp. 1, *G.* sp. 2, *G.* sp. 3, *G.* sp. 4, *G. symplocosis*	
	*Greenideoida*	*G. longirostrum*, *G. lutea*	2
	*Mesotrichosiphum*	*M. pentaiarticulatum*	1
	*Mollitrichosiphum*	*M. luchuanum*, *M. montanum*, *M. nandii*, *M. nigrofasciatum*, *M. nigrum*,	65
		*M. rhusae*, *M. tenuicorpus*, *M. tumorisiphum*	
Schoutedeniini	*Schoutedenia*	*S. emblica*, *S. ralumensis*	11

DNA extraction was performed using the whole body of a single adult viviparous female per colony. To remove external microbial contaminants, aphid individuals were initially washed with 70% ethanol for 5 min and then rinsed with sterile water five times. Total DNA was extracted using the DNeasy Blood & Tissue Kit (QIAGEN, Hilden, Germany). The negative control contained an equal amount of sterile water instead of aphid DNA. To identify aphid species and eliminate parasitized samples, DNA extracts were verified using National Center for Biotechnology Information (NCBI) BLAST searches of the barcode sequence targeting the cytochrome c oxidase subunit I (COI) gene. The DNA samples were kept at −20°C.

### 16S rRNA Gene Amplification, Illumina Sequencing, and Sequence Processing

The V3–V4 hypervariable region of the 16S rRNA gene was amplified using the primers 341F (5′-CCTAYGGGRBGCASCAG-3′) and 806R (5′-GGACTACNNGGGTATCTAAT-3′). A negative control was set up for the polymerase chain reaction (PCR), and all amplifications were conducted in triplicate. The 30-μL PCR was prepared with 15 μL of Phusion High-Fidelity PCR Master Mix (New England Biolabs, Ipswich, MA, United States), 3 μL primers and 10 ng of template DNA. The PCR thermal program was as follows: 1 min at 98°C for initial denaturation; 30 cycles of 10 s at 98°C for denaturing, 30 s at 50°C for annealing and 30 s at 72°C for elongation; and 5 min at 72°C for final extension.

Amplification products were recovered using 2% agarose gel electrophoresis, and positive samples with a bright band of 400–450 bp were purified with the GeneJET Gel Extraction Kit (Thermo Scientific, Wilmington, DE, United States). Sequencing libraries were constructed with the NEBNext Ultra DNA Library Prep Kit (New England Biolabs, Ipswich, MA, United States) and quantified by a Qubit 2.0 fluorometer (Thermo Scientific, Wilmington, DE, United States) and an Agilent Bioanalyzer 2100 system. Finally, the libraries were sequenced on an Illumina HiSeq 2500 PE250 platform (Illumina, San Diego, CA, United States).

Paired-end reads were assembled with a minimum overlap size of 10 bp and an error rate of 10% using FLASH v1.2.7 (Magoč and Salzberg, 2011). Low-quality sequences were filtered by QIIME v1.9.1 (Caporaso et al., 2010), and chimeras were discarded by UCHIME v4.2.40 (Edgar et al., 2011). Then, sequences were clustered into one operational taxonomic unit (OTU) at 97% similarity. Taxonomic annotations of OTUs were assessed with the RDP classifier (Wang et al., 2007) against the SILVA 128 reference database (Quast et al., 2013). We also manually checked the taxonomic affiliation using BLAST searches against the GenBank database. The OTUs with abundances below 0.005% were filtered as described by Bokulich et al. (2013). The mean sequence number of three PCR replicates per sample was calculated to estimate the abundance of each OTU. To mitigate the heterogeneity of sequencing depth, each sample was rarefied to the minimum value across all the samples (53,497 reads) using the ‘otutab_norm’ function in USEARCH v10.0 (Edgar, 2010). Finally, we obtained an OTU table containing taxonomic classifications of bacterial OTUs and the sequence number per sample (Supplementary Table 2a).

### Statistical Analyses

To better explore the variations in microbial community structures associated with Greenideinae, two reduced OTU tables, including symbionts (*Buchnera* and secondary symbionts) (Supplementary Table 2b) and secondary symbionts (Supplementary Table 2c), of aphids were generated. The relative abundances of OTUs were calculated using the *decostand* function and *total* method in the R package *vegan* (Oksanen et al., 2018). The distribution and relative abundances of symbionts were mapped against the phylogeny of Greenideinae aphids (detailed analysis methods are provided in the Supplementary Material) using iTOL v6.3.2 (Letunic and Bork, 2021). We implemented all of the subsequent statistical analyses with the bacterial, symbiont and secondary symbiont OTU tables. Samples were grouped according to aphid genus, aphid species, geographic distribution and host plant. Detailed grouping information is shown in Supplementary Table 3. Statistical analyses were conducted on all groups and groups with a sample size ≥ 3, excluding Mantel tests, Procrustes analyses, and ancestral secondary symbiont reconstruction. Samples with ambiguous information were excluded from analyses.

Shannon and Simpson indices were assessed to evaluate the alpha diversity using the *diversity* function of *vegan*. Considering the non-normal distribution (Shapiro–Wilk test, *p* < 0.05) of alpha diversity data, we used non-parametric Kruskal–Wallis tests to investigate the microbiota variation among groups. To simultaneously estimate the separate contributions of aphid genus (or aphid species), geography and host plant to microbial alpha diversity, three-way analysis of variance (ANOVA) was performed using the *avop* function in the *lmPerm* package (Wheeler and Torchiano, 2010). Three-way ANOVA was conducted on groups with sample size ≥ 2 and sample size ≥ 3, as the groups with one sample were insufficient for this analysis.

The Bray–Curtis distance (*vegdist* function of *vegan*) and unweighted UniFrac distance (*GUniFrac* function of *GUniFrac*) (Chen and Chen, 2018) were calculated to quantify beta diversity. First, we applied two kinds of ordination analyses, namely, unconstrained non-metric multidimensional scaling (NMDS) and constrained principal coordinate analysis (cPCoA), to visualize the dissimilarity of microbial communities based on the beta diversity. Based on Bray–Curtis and unweighted UniFrac distance matrices, NMDS (stress values < 0.05 indicate excellent representation) was performed with the *metaMDS* function in *vegan*. CPCoA (*capscal*e and *anova.cca* functions in *vegan*) was implemented with Bray–Curtis distance, as unweighted UniFrac distance was not suitable for this analysis. Then, the statistical significance of the microbiota variation between groups was determined by permutational multivariate analysis of variance (PERMANOVA) and analysis of similarities (ANOSIM). PERMANOVA and ANOSIM were conducted on Bray–Curtis and unweighted UniFrac distances using the *adonis* and *anosim* functions, respectively, with 10,000 permutations in *vegan*.

To explore the relationship between aphid phylogeny and microbiota dissimilarities, the Mantel test (*mantel* function) and Procrustes analysis (*procrustes* and *protest* functions) were performed with all samples using the matrices of aphid cophenetic distances and beta diversity (Bray–Curtis and unweighted UniFrac distances) in *vegan*. Cophenetic distances calculate the pairwise distances between the pairs of tips from a phylogenetic tree using its branch lengths. We used the *cophenetic* function in the package *ape* (Paradis and Schliep, 2019) to calculate the cophenetic distances of aphid samples. The Mantel test is a commonly used approach to quantify the correlation between two matrices (Anderson and Walsh, 2013). Procrustes analysis is more powerful, in which two matrices are scaled and rotated to maximize their similarity using principal component analysis (PCA) (Peres-Neto and Jackson, 2001).

To investigate the microbial composition changes that occurred during the evolutionary history of Greenideinae, a presence/absence matrix of six secondary symbionts in this study was mapped onto an aphid phylogenetic tree. The relationships of aphid species were simplified from the maximum-likelihood tree of Greenideinae aphid samples. Ancestral microbiome reconstruction was performed using parsimony and Bayesian approach. Parsimony reconstruction was implemented in Mesquite v3.70 (Maddison and Maddison, 2021) with the ‘trace character history’ option and unordered character state transformations. For Bayesian reconstruction, we applied the reverse jump Markov chain Monte Carlo framework with a prior drawn from a uniform distribution in BayesTraits V3.0.5 software (Pagel and Meade, 2006). Three independent analyses were performed for a total of 6,000,000 generations, sampling every 1000 generations. The first 25% of the generations were removed as burn-in, and the acceptance rates were between 0.2 and 0.4.

## Results

### Microbial Community Composition Across Greenideinae Aphids

After all quality filtering, a total of 11,506,428 reads (53,518 reads per sample) were obtained. The 16S rRNA gene sequences were clustered into 162 OTUs and annotated to 35 genera, 24 families, 16 orders, 12 classes, and 6 phyla of bacteria. At the phylum level, the microbial community of Greenideinae was composed mainly of Proteobacteria, with an average relative abundance of 98.74%. Gammaproteobacteria (95.15%) and Enterobacteriales (94.72%) were the most commonly classified class and order, respectively. The most abundant family was Enterobacteriaceae (94.42%), followed by Anaplasmataceae (2.10%) and Rickettsiaceae (1.34%). At the genus level, seven symbionts were detected, among which the relative abundances of *Buchnera* (68.19%), *S. symbiotica* (14.97%), *Wolbachia* (2.10%), *Arsenophonus* (1.80%), and *Rickettsia* (1.34%) were greater than 1% (Supplementary Table 4).

**FIGURE 1:**
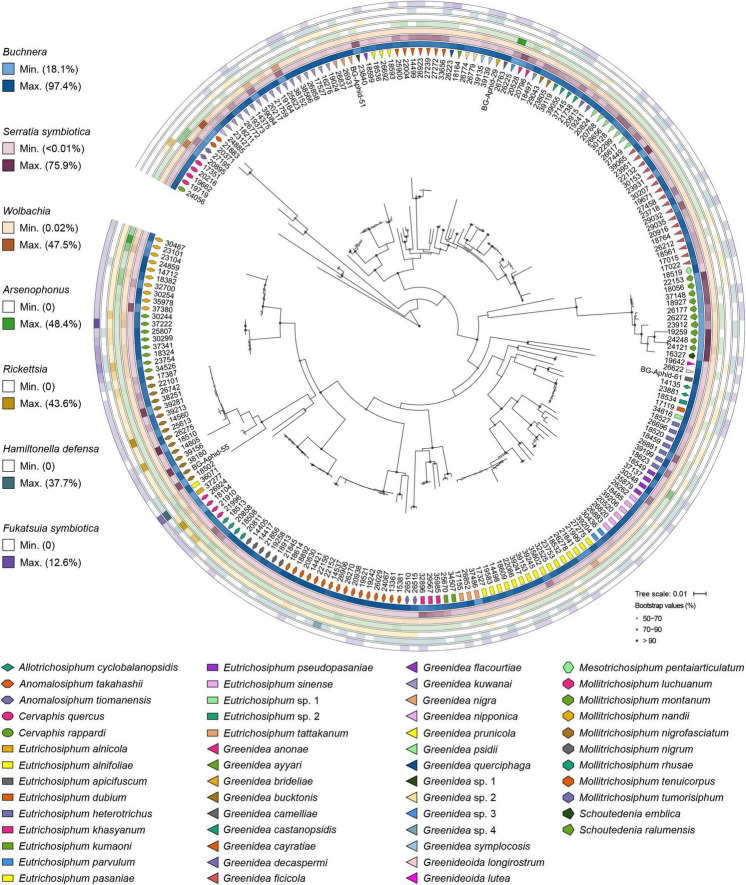
Symbiont community composition shown on the maximum-likelihood tree of Greenideinae aphids. Gray dots on the phylogeny nodes indicate bootstrap support of over 50%.

The primary endosymbiont *Buchnera* was present in all the samples and predominated in most. In addition, each examined sample was simultaneously infected with 2–6 secondary symbionts. *S. symbiotica* and *Wolbachia* were the most common, with an infection frequency of 215/215, followed by *Arsenophonus* (171/215), *Rickettsia* (96/215), *H. defensa* (88/215), and *F. symbiotica* (86/215). *S. symbiotica* was the most abundant secondary symbiont in Greenideinae, and its relative abundance was even higher than that of *Buchnera* in the aphid genus *Schoutedenia*. The infection pattern of secondary symbionts within Greenideinae was variable among different aphid genera and species (Figure 1). The most frequent secondary symbiont infection type was the combination of *S. symbiotica*, *Wolbachia*, and *Arsenophonus* (50/215) (Supplementary Table 5).

**FIGURE 2:**
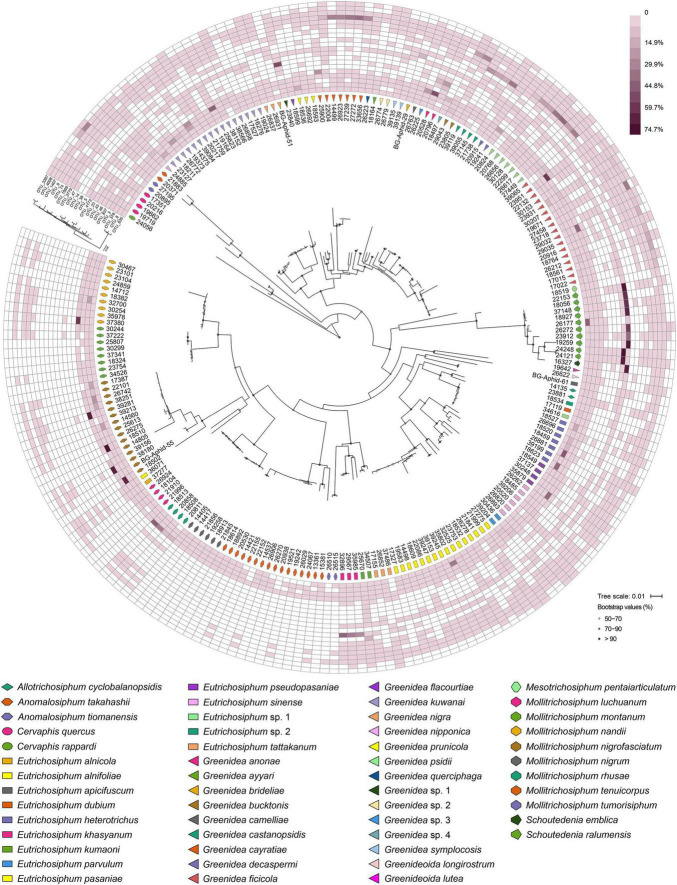
Heatmap representing the distribution and relative abundances of *Serratia symbiotica* OTUs among Greenideinae aphids. The phylogenetic relationships of Greenideinae and *S. symbiotica* OTUs based on maximum-likelihood analyses are presented.

**FIGURE 3:**
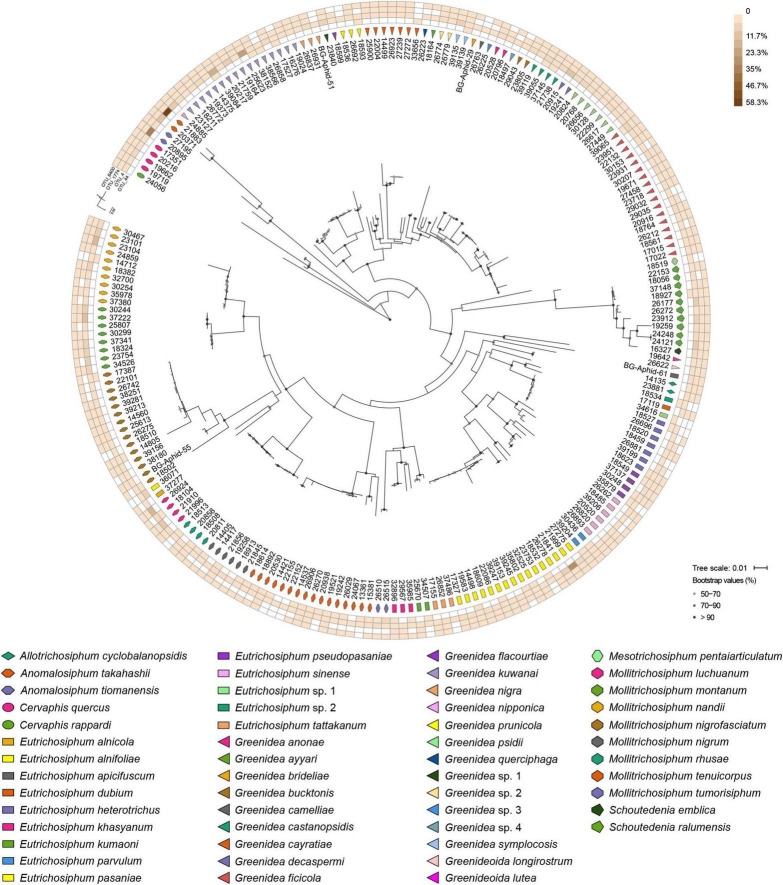
Heatmap representing the distribution and relative abundances of *Wolbachia* OTUs among Greenideinae aphids. The maximum-likelihood trees display the phylogenetic relationships of Greenideinae and *Wolbachia* OTUs.

At the OTU level, *Buchnera* (Supplementary Figure 1) and *S. symbiotica* (Figure 2) harbored 20–30 OTUs, whereas other secondary symbionts were represented by only 1–6 OTUs (Figure 3 and Supplementary Figures 2, 3). OTU11 or OTU 4915 of *Buchnera* predominated in most samples of the aphid genera *Cervaphis* and *Mollitrichosiphum* (Supplementary Figure 1). The aphid genera *Greenidea* and *Eutrichosiphum* were characterized mainly by more than one type of OTU belonging to *Buchnera*. Regarding the secondary symbionts, the dominant OTUs of *S. symbiotica* usually differed among aphid species, although most OTUs were widely distributed in Greenideinae (Figure 2). *Wolbachia* was represented by four OTUs, among which the relative abundance of OTU4 was high in some samples (Figure 3). We also observed the variability of the relative abundance and diversity of OTUs belonging to *Arsenophonus* associated with Greenideini species (excluding aphid species of *Anomalosiphum*, *Cervaphis*, and *Schoutedenia*) (Supplementary Figure 2). The prevalence and relative abundance of other secondary symbionts (i.e., *Rickettsia*, *H. defensa*, and *F. symbiotica*) were quite low in most samples (Supplementary Figure 3). For example, *H. defensa* and *Rickettsia* were not detected in the tribe Cervaphidini, which exhibited the lowest secondary symbiont diversity in the present study. The majority of Greenideinae aphids rarely host *Rickettsia*, except samples from *Mollitrichosiphum*.

### Variations in the Microbial Community Diversity of Greenideinae

Kruskal–Wallis tests of alpha diversity revealed a significant variation in the bacterial, symbiont and secondary symbiont communities among the aphid genera, aphid species and host plants (*P* < 0.05 for both Shannon and Simpson indices). A significant effect of geography on bacterial and symbiont communities was detected (Shannon, *P* = 0.014–0.040; Simpson, *n* ≥ 3, *P* = 0.021–0.027), except that there was no significant effect on the Simpson index upon using all samples (*P* = 0.057–0.066). However, the secondary symbiont communities did not differ significantly among geographic regions (*P* = 0.276–0.538).

Three-way ANOVA for alpha diversity indices highlighted the contribution of aphid identity (i.e., aphid genus or aphid species) to shaping the microbial community structures when the effect of different factors was estimated simultaneously. We found that the microbial communities differed significantly among aphid genera [Supplementary Table 6; *n* ≥ 2, *F*_(7_,_70)_ = 2.667–4.292, *P* ≤ 0.013; *n* ≥ 3, *F*_(4_,_69)_ = 5.333–7.833, *P* < 0.001] using all types of data in three-way ANOVA, whereas the effect of the host plant was not significant (*P* = 0.127–0.636). A significant effect of geography was found only on bacterial and symbiont communities with sample size ≥ 3 compared with the effect of aphid genus and host plant [*F*_(14_,_69)_ = 1.810–2.000, *P* = 0.005–0.027]. The results of three-way ANOVA also showed that the bacterial and symbiont communities were primarily structured by aphid species [Supplementary Table 7; *P* < 0.001; *n* ≥ 2, *F*_(28_,_38)_ = 9.333–11.375; *n* ≥ 3, *F*_(15_,_33)_ = 18.625–19.600]. Aphid species, geography and host plant did not have a significant impact on the secondary symbiont community (*P* = 0.174–0.924).

**FIGURE 4:**
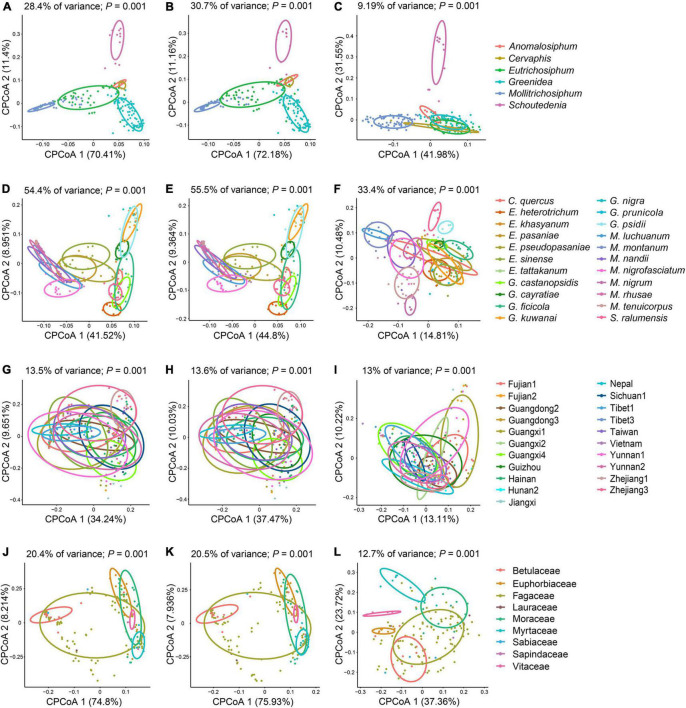
Constrained principal coordinate analysis (cPCoA) plots of Bray–Curtis distances of bacterial **(A,D,G,J)**, symbiont **(B,E,H,K)**, and secondary symbiont **(C,F,I,L)** communities (*n* ≥ 3). Plots are structured by aphid genus **(A–C)**, aphid species **(D–F)**, geographic region **(G–I)**, and host plant **(J–L)**.

Unconstrained NMDS plots based on all types of beta diversity matrices did not show meaningful clustering of samples structured by aphid genus, aphid species, geographic region or host plant (Supplementary Figures 4–7). Conversely, cPCoA plots of Bray–Curtis distances uncovered the significant patterns constrained by these four factors in bacterial, symbiont and secondary symbiont communities (Figure 4 and Supplementary Figure 8; *P* = 0.001). Aphid species (33.4–62.3% of variance) explained more of the overall variance in the data than aphid genus (9.19–32% of variance), geographic region (13–18.1% of variance) and host plant (12.7–26.9% of variance) in all the analyses.

PERMANOVA (*R*^2^ = 0.408–0.725, *P* < 0.001) and ANOSIM (*R* = 0.278–0.752, *P* < 0.001) corroborated the greatest effect of aphid species on structuring the microbial communities based on Bray–Curtis and unweighted UniFrac distances with sample size ≥ 3, although no statistical significance among aphid species (PERMANOVA: *P* = 0.108–0.141; ANOSIM: *P* = 0.127–0.391) or aphid genera (PERMANOVA: *P* = 0.726–0.778; ANOSIM: *P* = 0.386–0.548) was observed in most cases based on Bray–Curtis distances using all samples (Table 2). The significant impacts of geographic region (*P* ≤ 0.006) and host plant (*P* ≤ 0.047) were revealed by PERMANOVA using all types of beta diversity data. However, their contributions (geographic region: *R*^2^ = 0.145–0.643; host plant: *R*^2^ = 0.113–0.319) were usually limited compared to that of aphid species (*R*^2^ = 0.262–0.725). ANOSIM suggested significant dissimilarities in microbial communities among geographic regions using all samples (*R* = 0.325–0.576, *P* < 0.001). A significant effect of the host plant was found only in analyses of the Bray–Curtis distance (*R* = 0.135–0.177, *P* ≤ 0.001).

**TABLE 2:** Results of ANOSIM and PERMANOVA based on Bray–Curtis and unweighted Unifrac distances.

Beta diversity distance	Microbial community	Sample size	Aphid genus		Aphid species		Geographic region		Host plant	
			ANOSIM (*R*, *P*)	PERMANOVA (*R*^2^, *P*)	ANOSIM (*R*, *P*)	PERMANOVA (*R*^2^, *P*)	ANOSIM (*R*, *P*)	PERMANOVA (*R*^2^, *P*)	ANOSIM (*R*, *P*)	PERMANOVA (*R*^2^, *P*)
Bray–Curtis	Bacteria	*n* ≥ 1	0.001, 0.461	0.033, 0.726	0.030, 0.142	0.261, 0.108	0.349, <*0.001*	0.420, <*0.001*	0.135, <*0.001*	0.311, <*0.001*
		*n* ≥ 3	0.714, <*0.001*	0.438, <*0.001*	0.752, <*0.001*	0.713, <*0.001*	0.025, 0.165	0.150, <*0.001*	0.138, <*0.001*	0.214, <*0.001*
	Symbionts	*n* ≥ 1	0.004, 0.386	0.032, 0.778	0.031, 0.127	0.260, 0.141	0.325, <*0.001*	0.433, <*0.001*	0.139, <*0.001*	0.319, <*0.001*
		*n* ≥ 3	0.685, <*0.001*	0.466, <*0.001*	0.713, <*0.001*	0.725, <*0.001*	0.008, 0.358	0.151, *0.001*	0.148, <*0.001*	0.224, <*0.001*
	Secondary symbionts	*n* ≥ 1	–0.003, 0.548	0.035, 0.728	0.009, 0.391	0.262, *0.024*	0.374, <*0.001*	0.334, <*0.001*	0.145, *0.001*	0.253, <*0.001*
		*n* ≥ 3	0.297, <*0.001*	0.158, <*0.001*	0.555, <*0.001*	0.487, <*0.001*	0.033, 0.145	0.145, <*0.001*	0.177, <*0.001*	0.156, <*0.001*
Unweighted Unifrac	Bacteria	*n* ≥ 1	–0.010, 0.677	0.041, 0.293	–0.013, 0.652	0.256, 0.203	0.576, <*0.001*	0.519, <*0.001*	0.042, 0.187	0.246, <*0.001*
		*n* ≥ 3	0.377, <*0.001*	0.270, <*0.001*	0.469, <*0.001*	0.442, <*0.001*	0.085, *0.008*	0.183, <*0.001*	0.058, 0.122	0.146, <*0.001*
	Symbionts	*n* ≥ 1	0.057, *0.002*	0.052, 0.080	0.047, 0.056	0.292, *0.014*	0.520, <*0.001*	0.643, <*0.001*	–0.068, 0.966	0.207, *0.006*
		*n* ≥ 3	0.423, <*0.001*	0.377, <*0.001*	0.399, <*0.001*	0.530, <*0.001*	0.031, 0.139	0.151, *0.005*	–0.063, 0.939	0.116, *0.001*
	Secondary symbionts	*n* ≥ 1	0.053, *0.004*	0.062, *0.030*	0.066, *0.016*	0.327, *0.001*	0.415, <*0.001*	0.631, <*0.001*	–0.072, 0.978	0.208, *0.047*
		*n* ≥ 3	0.318, <*0.001*	0.281, <*0.001*	0.278, <*0.001*	0.408, <*0.001*	0.013, 0.328	0.156, *0.006*	–0.052, 0.904	0.113, *0.004*

*Statistically significant P-values (P < 0.05) are highlighted in italics.*

### Relationship Between Microbial Communities and Aphid Relatedness

**FIGURE 5:**
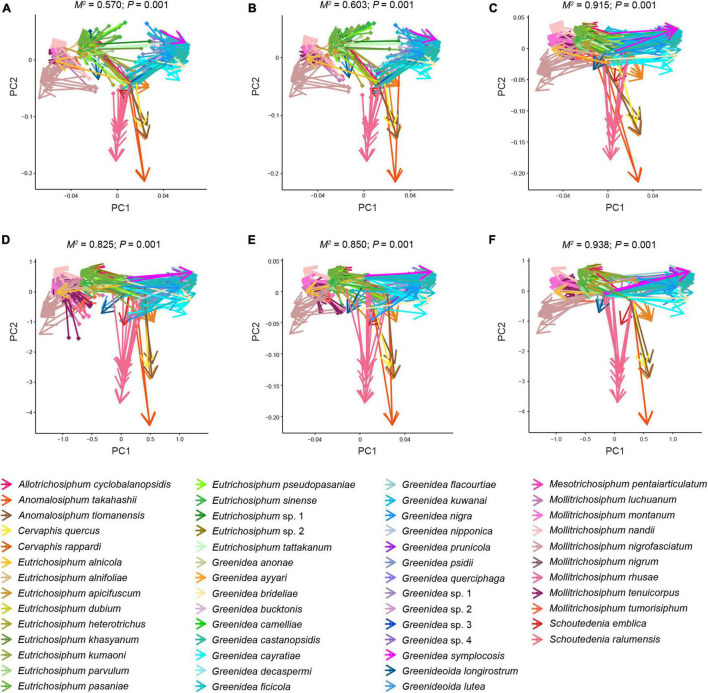
Procrustean superimpositions for PCA-scaled aphid cophenetic distances vs. variations in bacterial **(A,D)**, symbiont **(B,E)**, and secondary symbiont **(C,F)** communities. Bray–Curtis **(A–C)** and unweighted UniFrac distances **(D–F)** were used to estimate the microbiota variations.

Using the Mantel test with all types of beta diversity data, we detected a positively significant correlation between aphid phylogeny and the microbial profiles of bacterial, symbiont and secondary symbiont communities (Table 3; r = 0.145−0.555, *P* < 0.001). The results of Procrustes analysis were consistent, which showed that the microbial community structures were significantly related to aphid phylogeny (Figure 5; *M*^2^ = 0.570−0.938, *P* = 0.001).

**TABLE 3:** Correlations between microbial beta diversity and aphid phylogeny estimated by the Mantel test.

Microbial community	Beta diversity distance	Mantel test	
		*r*	*P*
Bacteria	Bray–Curtis	0.555	<*0.001*
	Unweighted Unifrac	0.234	<*0.001*
Symbionts	Bray–Curtis	0.531	<*0.001*
	Unweighted Unifrac	0.249	<*0.001*
Secondary symbionts	Bray–Curtis	0.243	<*0.001*
	Unweighted Unifrac	0.145	<*0.001*

*Statistically significant P-values (P < 0.05) are highlighted in italics.*

### Ancient Aphid-Secondary Symbiont Associations

**FIGURE 6:**
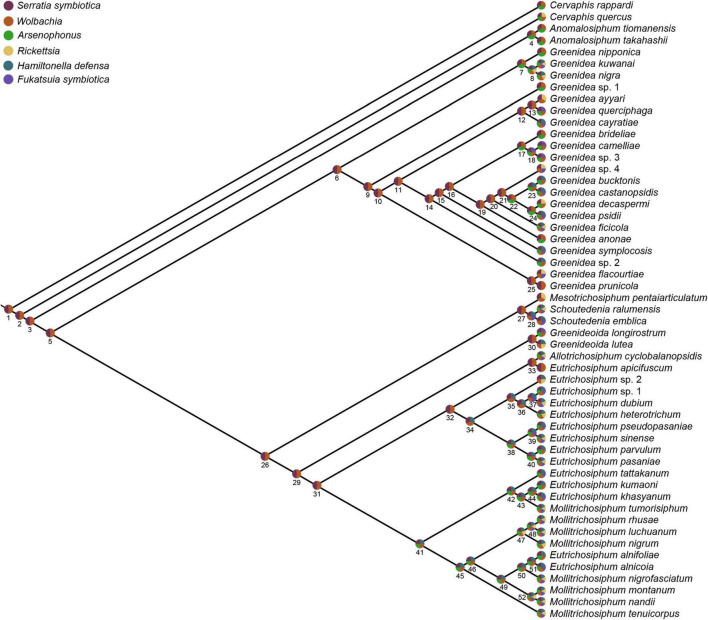
Ancestral associations of secondary symbionts and Greenideinae aphid species estimated by Bayesian reconstruction. Pie charts at nodes and tips show the presence of different secondary symbionts. The mean posterior probabilities at nodes are given in Supplementary Table 8.

The ancestral microbiome state was reconstructed to further understand shifts in the secondary symbionts over the evolutionary history of Greenideinae. Both parsimony and Bayesian analyses supported the acquisition of *S. symbiotica* and *Wolbachia* at the base of Greenideinae (Supplementary Figure 9; Figure 6, and Supplementary Table 8). Regarding the associations of other secondary symbionts and Greenideinae aphids, some incongruence was observed between different analysis approaches. The parsimony-based inference suggested that *Arsenophonus* was acquired by the most recent common ancestor of Greenideinae and subsequently lost in several aphid species. In addition, parsimony reconstruction reflected the separate gains of secondary symbionts in different aphid clades, such as *Rickettsia* in the clade containing *Mesotrichosiphum*, *Schoutedenia*, *Greenideoida*, *Allotrichosiphum*, *Eutrichosiphum*, and *Mollitrichosiphum* (Supplementary Figure 9). The results of Bayesian ancestral state reconstruction are summarized in Supplementary Table 8 and visualized in Figure 6; the results indicated two independent acquisition events for *H. defensa* in the clade containing *Eutrichosiphum* and *Mollitrichosiphum*.

## Discussion

### Symbiont Diversity Associated With Greenideinae Aphids

The microbial community composition of Greenideinae was dominated by aphid symbionts, among which seven symbionts were identified in the ten most abundant genera. The ubiquity and relative abundance of *Buchnera* substantiated its obligate nutritive role in the long-term endosymbiotic relationship with host aphids (Munson et al., 1991; Douglas, 1998; Liu et al., 2013; Xu et al., 2018). Multiple infections (i.e., infections with more than one type of symbiont in a host individual) of secondary symbionts were common in Greenideinae aphids. Positive interactions related to cohabitation of secondary symbionts have been demonstrated in many studies, such as higher resistance to parasitism than to single infection (Oliver et al., 2006). However, some coinfecting secondary symbionts with no conditional mutualism can persist in aphids by hitchhiking with beneficial symbionts (Smith et al., 2015).

*Serratia symbiotica* and *Wolbachia* were found in all examined Greenideinae samples with high relative abundance. Our study confirmed the widespread distribution of *Wolbachia* in aphids reported by Wang et al. (2014). It is worth noting that the relative abundance of *S. symbiotica* was higher than that of *Buchnera* in all samples of the aphid genus *Schoutedenia*. Rübsaamen (1905) has documented that *Schoutedenia* can induce galls on its host plants, differing greatly from other aphids in Greenideinae. This distinctive biological trait may give rise to different symbiont community structures and even novel nutrition-biosynthetic pathways provided by symbionts in the host *Schoutedenia.* The contribution of *S. symbiotica* to nutritional biosynthesis has been reported in some aphids (Lamelas et al., 2011; Mccutcheon and Moran, 2012; Bennett and Moran, 2015). Further research is required to illustrate the exact effects of *S. symbiotica* associated with *Schoutedenia* aphids.

*Arsenophonus* associated with Greenideini species exhibited various infection patterns at the OTU level. Liu et al. (2015) suggested that the acquisition of new host plants might account for species differentiation in Greenideini. Improvement of performance on novel host plants and other ecological benefits have been demonstrated in previous studies of *Arsenophonus* (Wagner et al., 2015; Lenhart and White, 2020). However, further experiments should be performed to determine whether *Arsenophonus* infection is related to the broadening of the host plant range in Greenideini.

Other secondary symbionts (i.e., *Rickettsia*, *H. defensa*, and *F. symbiotica*) were not frequent symbiotic partners, and their relative abundances were quite low in most samples. Aphid species in Greenideinae usually move rapidly and have long siphunculi that enable them to efficiently emit alarm pheromones (Mondor et al., 2002). In addition, ant attending was observed in many Greenideinae species (Blackman and Eastop, 2021), providing defense from natural enemies for aphids. Prior studies have reported the trade-off between fitness costs (e.g., reduced lifespan and fecundity) and benefits of hosting symbionts (Oliver et al., 2014; Leybourne et al., 2020; Zytynska et al., 2021). Therefore, aphids generally tend not to host defensive symbionts when they obtain protection from ecological habits (Henry et al., 2015). Greenideinae may have defensive effects derived from these life history traits, reducing the maintenance of secondary symbionts providing similar resistance to parasitism, such as *Rickettsia*, *H. defensa*, and *F. symbiotica*.

### Contribution of Different Factors to Shaping the Microbiota of Greenideinae

The results of alpha and beta diversity analyses highlighted the role of host identity in shaping the structures of bacterial, symbiont, and secondary symbiont communities associated with Greenideinae. We revealed the strongest impact of aphid species on microbial profiles, which was in line with previous evidence for the existence of a species-specific microbiota in *Mollitrichosiphum* aphids (Qin et al., 2021a). In addition, significant microbial community structures constrained by the aphid genus were identified. Acquiring a large amount of species-level taxonomic information on insect specimens is usually time-consuming and challenging. Our results suggest that genus-level insect identification is meaningful for investigating host-associated microbiota structures with limited taxonomic knowledge.

Prior studies have demonstrated that geography (Tsuchida et al., 2002; Guo et al., 2019; Xu S. F. et al., 2020) and host plant (Brady et al., 2014; Gauthier et al., 2015; Xu T. T. et al., 2020) are important factors structuring the symbiont community composition associated with aphids. Distinct environmental conditions (Sepúlveda et al., 2017) and spatial limitations on bacterial dispersal (Moeller et al., 2017) can lend to the variability of symbionts. In the present study, the geography and host plant significantly influenced the microbial profiles across Greenideinae, although their contributions were usually weaker than that of host identity. Symbiont-assisted speciation has been reviewed by Brucker and Bordenstein (2012). Some secondary symbionts of aphids, such as *Arsenophonus* (Wagner et al., 2015), play a crucial role in the exploitation and specialization of host plants. We observed OTU variability in *Arsenophonus* among Greenideini species. The species differentiation of Greenideini was involved in the enhancement of host plant utilization (Liu et al., 2015). Hence, it seems likely that secondary symbionts mediate dietary breadth in Greenideini, which potentially facilitates the diversification of aphids.

### Phylosymbiosis Between Greenideinae Aphids and Their Microbiota

The pattern of phylosymbiosis revealed in Greenideinae was characterized by a positively significant correlation between aphid phylogeny and microbial community dissimilarities. Mantel tests and Procrustes analyses using all types of data consistently showed the phylosymbiotic microbiota assemblage in bacterial, symbiont and secondary symbiont communities. Phylosymbiosis has been reported in host taxa with evolutionary histories ranging from approximately 0.3–108 million years (Brooks et al., 2016). However, several factors are likely to erode phylosymbiosis signals in natural populations during long evolutionary periods, such as horizontal transmission of microbes, complete dietary transition and changes in environmental filters. Combined with previous studies of *Mollitrichosiphum* (Qin et al., 2021a) and *M. tenicroperus* (Qin et al., 2021b), our studies revealed that phylosymbiosis can be observed at the subfamily, genus and species levels within natural populations of specific aphid groups, spanning recent host speciation events to more distant host divergence.

Considering the strictly maternal inheritance of *Buchnera* and primarily vertical transmission of some secondary symbionts in aphids, one of the possible mechanisms underlying phylosymbiosis is evolutionary processes, such as codiversification of Greenideinae and symbionts. The ancestral association between Greenideinae and two secondary symbionts, *S. symbiotica* and *Wolbachia*, was supported by ancestral microbiome reconstruction based on both parsimony and Bayesian approaches. The diversification tendency of *S. symbiotica* at the OTU level also indicated host-symbiont codiversification within Greenideinae. In this study, *Buchnera*, *S. symbiotica* and *Wolbachia* may serve as hub microbes, allowing determination of the whole microbial community composition through microbe–microbe interactions (Fisher and Mehta, 2014; Agler et al., 2016). Furthermore, previous studies did not detect a correlation between microbiota similarities and aphid phylogeny in heteroecious Eriosomatinae (Xu T. T. et al., 2020). The frequent horizontal transmission of secondary symbionts might have erased the phylosymbiosis signals in this aphid subfamily. In contrast, the monoecious life cycle of Greenideinae could have reduced the horizontal transmission of secondary symbionts among aphid populations colonizing different host plants. Therefore, codiversification of key symbionts and relatively stable microbial community composition may be responsible for the phylosymbiosis of Greenideinae.

Ecological filtering by conserved host traits (Moran and Sloan, 2015; Mazel et al., 2018) can also contribute to the mechanisms underpinning phylosymbiosis. Closely related hosts tend to harbor a similar microbiota owing to similar physiologies or immune responses regulating microbes. In addition, diet is an important filter shaping the host-associated microbiota, and complete dietary shifts over long evolutionary periods can even disrupt phylogenetic signals in microbial communities (Muegge et al., 2011; Groussin et al., 2017; Chiarello et al., 2018). Beta diversity analyses showed the influence of host plants on the structures of the microbial communities associated with Greenideinae. Dietary shifts, including direct switching to novel host plants and expansion of the host plant range, did not cover the phylosymbiosis signals in the present study. Host plants might be filters that are phylogenetically correlated with aphids, considering their promotion of species differentiation in Greenideinae. It seems reasonable that filtering by host plants is another driver yielding the phylosymbiotic microbiota of Greenideinae.

## Conclusion

Unraveling the influence of ecological and evolutionary factors on microbial community assembly is crucial to understanding symbiosis in nature. We provided the first systematic microbiota landscape associated with Greenideinae. The significant impacts of host identity, geography and host plant on microbial community structures were uncovered, among which the primary contribution of aphid species was highlighted. Moreover, our study found that microbial community dissimilarities are correlated with aphid relatedness, providing evidence for phylosymbiosis in natural aphid populations. We propose that phylosymbiosis in Greenideinae relies on multiple mechanisms. Aphid-symbiont codiversification and filtering by host plants might contribute to the phylosymbiotic microbiota in Greenideinae.

## Data Availability Statement

The datasets presented in this study can be found in online repositories. The names of the repository/repositories and accession number(s) can be found below: GenBank under accession numbers OL619437–OL619530, OL624544–OL624640, and OL631781–OL631898. Raw 16S rRNA gene amplicon reads were deposited in the NCBI Sequence Read Archive under BioProject accession number PRJNA783284 and PRJNA637573.

## Author Contributions

GQ and LJ designed the project. GQ, JC, and LJ identified voucher specimens. MQ conducted molecular experiments and all analyses and wrote the manuscript. JC assisted data analyses. All the authors contributed to revisions.

## Conflict of Interest

The authors declare that the research was conducted in the absence of any commercial or financial relationships that could be construed as a potential conflict of interest.

## Publisher’s Note

All claims expressed in this article are solely those of the authors and do not necessarily represent those of their affiliated organizations, or those of the publisher, the editors and the reviewers. Any product that may be evaluated in this article, or claim that may be made by its manufacturer, is not guaranteed or endorsed by the publisher.

## References

[B1] Agler M. T. Ruhe J. Kroll S. Morhenn C. Kim S. Weigel D. (2016). Microbial hub taxa link host and abiotic factors to plant microbiome variation. *PLoS Biol.* 14:e1002352. 10.1371/journal.pbio.1002352 26788878 PMC4720289

[B2] Anderson M. J. Walsh D. C. (2013). PERMANOVA, ANOSIM, and the mantel test in the face of heterogeneous dispersions: what null hypothesis are you testing? *Ecol. Monogr.* 83 557–574. 10.1890/12-2010.1

[B3] Augustinos A. A. Santos-Garcia D. Dionyssopoulou E. Moreira M. Papapanagiotou A. Scarvelakis M. (2011). Detection and characterization of *Wolbachia* infections in natural populations of aphids: is the hidden diversity fully unraveled? *PLoS One* 6:e28695. 10.1371/journal.pone.0028695 22174869 PMC3236762

[B4] Baumann P. Baumann L. Lai C. Y. Rouhbakhsh D. Moran N. A. Clark M. A. (1995). Genetics, physiology, and evolutionary relationships of the genus *Buchnera*: intracellular symbionts of aphids. *Annu. Rev. Microbiol.* 49 55–94. 10.1146/annurev.mi.49.100195.000415 8561471

[B5] Bennett G. M. Moran N. A. (2015). Heritable symbiosis: the advantages and perils of an evolutionary rabbit hole. *Proc. Natl. Acad. Sci. U.S.A.* 112 10169–10176. 10.1073/pnas.1421388112 25713367 PMC4547261

[B6] Blackman R. L. Eastop V. F. (2021). *Aphids on the World’s Plants: an Online Identification and Information Guide.* Available Online at: http://www.aphidsonworldsplants.info [accessed October 8, 2021].

[B7] Bokulich N. A. Subramanian S. Faith J. J. Gevers D. Gordon J. I. Knight R. (2013). Quality-filtering vastly improves diversity estimates from Illumina amplicon sequencing. *Nat. Methods* 10 57–59. 10.1038/nmeth.2276 23202435 PMC3531572

[B8] Brady C. M. Asplen M. K. Desneux N. Heimpel G. E. Hopper K. R. Linnen C. R. (2014). Worldwide populations of the aphid *Aphis craccivora* are infected with diverse facultative bacterial symbionts. *Microb. Ecol.* 67 195–204. 10.1007/s00248-013-0314-0 24233285

[B9] Brady C. M. White J. A. (2013). Cowpea aphid (*Aphis craccivora*) associated with different host plants has different facultative endosymbionts. *Ecol. Entomol.* 38 433–437. 10.1111/een.12020

[B10] Brooks A. W. Kohl K. D. Brucker R. M. van Opstal E. J. Bordenstein S. R. (2016). Phylosymbiosis: relationships and functional effects of microbial communities across host evolutionary history. *PLoS Biol.* 14:e2000225. 10.1371/journal.pbio.2000225 27861590 PMC5115861

[B11] Brucker R. M. Bordenstein S. R. (2012). Speciation by symbiosis. *Trends Ecol. Evol.* 27 443–451. 10.1016/j.tree.2012.03.011 22541872

[B12] Brucker R. M. Bordenstein S. R. (2013). The hologenomic basis of speciation: gut bacteria cause hybrid lethality in the genus *Nasonia*. *Science* 341 667–669. 10.1126/science.1240659 23868918

[B13] Caporaso J. G. Kuczynski J. Stombaugh J. Bittinger K. Bushman F. D. Costello E. K. (2010). QIIME allows analysis of high-throughput community sequencing data. *Nat. Methods* 7 335–336. 10.1038/nmeth.f.303 20383131 PMC3156573

[B14] Chen D. Q. Campbell B. C. Purcell A. H. (1996). A new *Rickettsia* from a herbivorous insect, the pea aphid *Acyrthosiphon pisum* (Harris). *Curr. Microbiol.* 33 123–128. 10.1007/s002849900086 8662184

[B15] Chen D. Q. Montllor C. B. Purcell A. H. (2000). Fitness effects of two facultative endosymbiotic bacteria on the pea aphid, *Acyrthosiphon pisum*, and the blue alfalfa aphid, *A. kondoi*. *Entomol. Exp. Appl.* 95 315–323. 10.1046/j.1570-7458.2000.00670.x

[B16] Chen J. Chen M. J. (2018). *Package ‘GUniFrac’. R Package Version 1.1.* Available Online at: https://CRAN.R-project.org/package=GUniFrac [accessed June 18, 2020].

[B17] Chiarello M. Auguet J. Bettarel Y. Bouvier C. Claverie T. Graham N. A. (2018). Skin microbiome of coral reef fish is highly variable and driven by host phylogeny and diet. *Microbiome* 6:147. 10.1186/s40168-018-0530-4 30143055 PMC6109317

[B18] Clark M. A. Moran N. A. Baumann P. Wernegreen J. J. (2000). Cospeciation between bacterial endosymbionts (*Buchnera*) and a recent radiation of aphids (*Uroleucon*) and pitfalls of testing for phylogenetic congruence. *Evolution* 54 517–525. 10.1111/j.0014-3820.2000.tb00054.x 10937228

[B19] Darby A. C. Birkle L. M. Turner S. L. Douglas A. E. (2001). An aphid-borne bacterium allied to the secondary symbionts of whitefly. *FEMS Microbiol. Ecol.* 36 43–50. 10.1016/S0168-6496(01)00117-9 11377772

[B20] Douglas A. E. (1998). Nutritional interactions in insect-microbial symbioses: aphids and their symbiotic bacteria *Buchnera*. *Annu. Rev. Entomol.* 43 17–37. 10.1146/annurev.ento.43.1.17 15012383

[B21] Edgar R. C. (2010). Search and clustering orders of magnitude faster than BLAST. *Bioinformatics* 26 2460–2461. 10.1093/bioinformatics/btq461 20709691

[B22] Edgar R. C. Haas B. J. Clemente J. C. Quince C. Knight R. (2011). UCHIME improves sensitivity and speed of chimera detection. *Bioinformatics* 27 2194–2200. 10.1093/bioinformatics/btr381 21700674 PMC3150044

[B23] Favret C. (2021). *Aphid Species File.* Available Online at: http://Aphid.SpeciesFile.org [accessed October 8, 2021].

[B24] Ferrari J. West J. A. Via S. Godfray H. C. J. (2012). Population genetic structure and secondary symbionts in host-associated populations of the pea aphid complex. *Evolution* 66 375–390. 10.1111/j.1558-5646.2011.01436.x 22276535

[B25] Fisher C. K. Mehta P. (2014). Identifying keystone species in the human gut microbiome from metagenomic timeseries using sparse linear regression. *PLoS One* 9:e102451. 10.1371/journal.pone.0102451 25054627 PMC4108331

[B26] Fukatsu T. Nikoh N. Kawai R. Koga R. (2000). The secondary endosymbiotic bacterium of the pea aphid *Acyrthosiphon pisum* (Insecta: Homoptera). *Appl. Environ. Microbiol.* 66 2748–2758. 10.1128/AEM.66.7.2748-2758.2000 10877764 PMC92069

[B27] Fukatsu T. Tsuchida T. Nikoh N. Koga R. (2001). *Spiroplasma* symbiont of the pea aphid, *Acyrthosiphon pisum* (Insecta: Homoptera). *Appl. Environ. Microbiol.* 67 1284–1291. 10.1128/AEM.67.3.1284-1291.2001 11229923 PMC92726

[B28] Gallo-Franco J. J. Duque-Gamboa D. N. Toro-Perea N. (2019). Bacterial communities of *Aphis gossypii* and *Myzus persicae* (Hemiptera: Aphididae) from pepper crops (*Capsicum* sp.). *Sci. Rep.* 9:5766. 10.1038/s41598-019-42232-8 30962510 PMC6453963

[B29] Gauthier J. P. Outreman Y. Mieuzet L. Simon J. C. (2015). Bacterial communities associated with host-adapted populations of pea aphids revealed by deep sequencing of 16S ribosomal DNA. *PLoS One* 10:e0120664. 10.1371/journal.pone.0120664 25807173 PMC4373712

[B30] Groussin M. Mazel F. Sanders J. G. Smillie C. S. Lavergne S. Thuiller W. (2017). Unraveling the processes shaping mammalian gut microbiomes over evolutionary time. *Nat. Commun.* 8:14319. 10.1038/ncomms14319 28230052 PMC5331214

[B31] Guay J. F. Boudreault S. Michaud D. Cloutier C. (2009). Impact of environmental stress on aphid clonal resistance to parasitoids: role of *Hamiltonella defensa* bacterial symbiosis in association with a new facultative symbiont of the pea aphid. *J. Insect Physiol.* 55 919–926. 10.1016/j.jinsphys.2009.06.006 19545573

[B32] Guo J. Q. Liu X. W. Poncelet N. He K. L. Francis F. Wang Z. Y. (2019). Detection and geographic distribution of seven facultative endosymbionts in two *Rhopalosiphum* aphid species. *Microbiologyopen* 8:e00817. 10.1002/mbo3.817 30912316 PMC6692527

[B33] Guyomar C. Legeai F. Jousselin E. Mougel C. Lemaitre C. Simon J. (2018). Multi-scale characterization of symbiont diversity in the pea aphid complex through metagenomic approaches. *Microbiome* 6:181. 10.1186/s40168-018-0562-9 30305166 PMC6180509

[B34] Hammer T. J. Dickerson J. C. McMillan W. O. Fierer N. (2020). *Heliconius* butterflies host characteristic and phylogenetically structured adult-stage microbiomes. *Appl. Environ. Microbiol.* 86:e02007-20. 10.1128/AEM.02007-20 33008816 PMC7688219

[B35] Henry L. M. Maiden M. C. Ferrari J. Godfray H. C. J. (2015). Insect life history and the evolution of bacterial mutualism. *Ecol. Lett.* 18 516–525. 10.1111/ele.12425 25868533

[B36] Heyworth E. R. Ferrari J. (2015). A facultative endosymbiont in aphids can provide diverse ecological benefits. *J. Evol. Biol.* 28 1753–1760. 10.1111/jeb.12705 26206380 PMC4949989

[B37] Jousselin E. Clamens A. L. Galan M. Bernard M. Maman S. Gschloessl B. (2016). Assessment of a 16S rRNA amplicon Illumina sequencing procedure for studying the microbiome of a symbiont-rich aphid genus. *Mol. Ecol. Resour.* 16 628–640. 10.1111/1755-0998.12478 26458227

[B38] Koga R. Meng X. Tsuchida T. Fukatsu T. (2012). Cellular mechanism for selective vertical transmission of an obligate insect symbiont at the bacteriocyte-embryo interface. *Proc. Natl. Acad. Sci. U.S.A.* 109 E1230–E1237. 10.1073/pnas.1119212109 22517738 PMC3356617

[B39] Lamelas A. Gosalbes M. J. Manzano-Marín A. Peretó J. Moya A. Latorre A. (2011). *Serratia symbiotica* from the aphid *Cinara cedri*: a missing link from facultative to obligate insect endosymbiont. *PLoS Genet.* 7:e1002357. 10.1371/journal.pgen.1002357 22102823 PMC3213167

[B40] Lenhart P. A. White J. A. (2020). Endosymbionts facilitate rapid evolution in a polyphagous herbivore. *J. Evol. Biol.* 33 1507–1511. 10.1111/jeb.13697 32894786

[B41] Letunic I. Bork P. (2021). Interactive tree of life (iTOL) v5: an online tool for phylogenetic tree display and annotation. *Nucleic Acids Res.* 49 W293–W296. 10.1093/nar/gkab301 33885785 PMC8265157

[B42] Leybourne D. J. Bos J. I. Valentine T. A. Karley A. J. (2020). The price of protection: a defensive endosymbiont impairs nymph growth in the bird cherry-oat aphid, *Rhopalosiphum padi*. *Insect Sci.* 27 69–85. 10.1111/1744-7917.12606 29797656 PMC7379937

[B43] Lim S. J. Bordenstein S. R. (2020). An introduction to phylosymbiosis. *Proc. R. Soc. B Biol. Sci.* 287:20192900. 10.1098/rspb.2019.2900 32126958 PMC7126058

[B44] Liu L. Huang X. L. Zhang R. L. Jiang L. Y. Qiao G. X. (2013). Phylogenetic congruence between *Mollitrichosiphum* (Aphididae: Greenideinae) and *Buchnera* indicates insect-bacteria parallel evolution. *Syst. Entomol.* 38 81–92. 10.1111/j.1365-3113.2012.00647.x

[B45] Liu Q. H. Chen J. Huang X. L. Jiang L. Y. Qiao G. X. (2015). Ancient association with Fagaceae in the aphid tribe Greenideini (Hemiptera: Aphididae: Greenideinae). *Syst. Entomol.* 40 230–241. 10.1111/syen.12100

[B46] Liu X. D. Lei H. X. Chen F. F. (2019). Infection pattern and negative effects of a facultative endosymbiont on its insect host are environment-dependent. *Sci. Rep.* 9:4013. 10.1038/s41598-019-40607-5 30850675 PMC6408509

[B47] Maddison W. P. Maddison D. R. (2021). *Mesquite: a Modular System for Evolutionary Analysis. Version 3.70.* Available Online at: http://www.mesquiteproject.org [accessed August 1, 2021].

[B48] Magoč T. Salzberg S. L. (2011). FLASH: fast length adjustment of short reads to improve genome assemblies. *Bioinformatics* 27 2957–2963. 10.1093/bioinformatics/btr507 21903629 PMC3198573

[B49] Mazel F. Davis K. M. Loudon A. Kwong W. K. Groussin M. Parfrey L. W. (2018). Is host filtering the main driver of phylosymbiosis across the tree of life? *mSystems* 3:e00097-18. 10.1128/mSystems.00097-18 30417109 PMC6208643

[B50] Mccutcheon J. P. Moran N. A. (2012). Extreme genome reduction in symbiotic bacteria. *Nat. Rev. Microbiol.* 10 13–26. 10.1038/nrmicro2670 22064560

[B51] McLean A. H. C. Godfray H. C. J. Ellers J. Henry L. M. (2019). Host relatedness influences the composition of aphid microbiomes. *Environ. Microbiol. Rep.* 11 808–816. 10.1111/1758-2229.12795 31573138 PMC6900097

[B52] Michalik A. Szklarzewicz T. Jankowska W. Wieczorek K. (2014). Endosymbiotic microorganisms of aphids (Hemiptera: Sternorrhyncha: Aphidoidea): ultrastructure, distribution and transovarial transmission. *Eur. J. Entomol.* 111 91–104. 10.14411/eje.2014.011

[B53] Moeller A. H. Suzuki T. A. Lin D. Lacey E. A. Wasser S. K. Nachman M. W. (2017). Dispersal limitation promotes the diversification of the mammalian gut microbiota. *Proc. Natl. Acad. Sci. U.S.A.* 114 13768–13773. 10.1073/pnas.1700122114 29229828 PMC5748161

[B54] Mondor E. B. Roitberg B. D. Stadler B. (2002). Cornicle length in macrosiphini aphids: a comparison of ecological traits. *Ecol. Entomol.* 27 758–762. 10.1046/j.1365-2311.2002.00470.x

[B55] Montllor C. B. Maxmen A. Purcell A. H. (2002). Facultative bacterial endosymbionts benefit pea aphids *Acyrthosiphon pisum* under heat stress. *Ecol. Entomol.* 27 189–195. 10.1046/j.1365-2311.2002.00393.x

[B56] Moran N. A. Sloan D. B. (2015). The hologenome concept: helpful or hollow? *PLoS Biol.* 13:e1002311. 10.1371/journal.pbio.1002311 26636661 PMC4670207

[B57] Muegge B. D. Kuczynski J. Knights D. Clemente J. C. Gonzalez A. Fontana L. (2011). Diet drives convergence in gut microbiome functions across mammalian phylogeny and within humans. *Science* 332 970–974. 10.1126/science.1198719 21596990 PMC3303602

[B58] Munson M. A. Baumann P. Clark M. A. Baumann L. Moran N. A. Voegtlin D. J. (1991). Evidence for the establishment of aphid-eubacterium endosymbiosis in an ancestor of four aphid families. *J. Bacteriol.* 173 6321–6324. 10.1128/jb.173.20.6321-6324.1991 1917864 PMC208962

[B59] Oksanen J. Blanchet F. G. Friendly M. Kindt R. Legendre P. McGlinn D. (2018). *Package ‘Vegan’, Community Ecology Package. Version 2.5-2.* Available Online at: https://cran.r-project.org/src/contrib/Archive/vegan/ [accessed June 18, 2021].

[B60] Oliver K. M. Moran N. A. Hunter M. S. (2006). Costs and benefits of a superinfection of facultative symbionts in aphids. *Proc. R. Soc. B Biol. Sci.* 273 1273–1280. 10.1098/rspb.2005.3436 PMC1560284 16720402

[B61] Oliver K. M. Russell J. A. Moran N. A. Hunter M. S. (2003). Facultative bacterial symbionts in aphids confer resistance to parasitic wasps. *Proc. Natl. Acad. Sci. U.S.A.* 100 1803–1807. 10.1073/pnas.0335320100 12563031 PMC149914

[B62] Oliver K. M. Smith A. H. Russell J. A. (2014). Defensive symbiosis in the real world – advancing ecological studies of heritable, protective bacteria in aphids and beyond. *Funct. Ecol.* 28 341–355. 10.1111/1365-2435.12133

[B63] Pagel M. Meade A. (2006). Bayesian analysis of correlated evolution of discrete characters by reversible-jump Markov Chain Monte Carlo. *Am. Nat.* 167 808–825. 10.1086/503444 16685633

[B64] Paradis E. Schliep K. (2019). ape 5.0: an environment for modern phylogenetics and evolutionary analyses in R. *Bioinformatics* 35 526–528. 10.1093/bioinformatics/bty633 30016406

[B65] Peres-Neto P. R. Jackson D. A. (2001). How well do multivariate data sets match? The advantages of a procrustean superimposition approach over the mantel test. *Oecologia* 129 169–178. 10.1007/s004420100720 28547594

[B66] Pons I. Renoz F. Noël C. Hance T. (2019). Circulation of the cultivable symbiont *Serratia symbiotica* in aphids is mediated by plants. *Front. Microbiol.* 10:764. 10.3389/fmicb.2019.00764 31037067 PMC6476230

[B67] Qin M. Chen J. Xu S. F. Jiang L. Y. Qiao G. X. (2021a). Microbiota associated with *Mollitrichosiphum* aphids (Hemiptera: Aphididae: Greenideinae): diversity, host species specificity and phylosymbiosis. *Environ. Microbiol.* 23 2184–2198. 10.1111/1462-2920.15391 33415800 PMC8248049

[B68] Qin M. Jiang L. Y. Kholmatov B. R. Qiao G. X. Chen J. (2021b). Phylosymbiotic structures of the microbiota in *Mollitrichosiphum tenuicorpus* (Hemiptera: Aphididae: Greenideinae). *Microb. Ecol.* [Online ahead of print]. 10.1007/s00248-021-01830-8 PMC9250915 34387702

[B69] Quast C. Pruesse E. Yilmaz P. Gerken J. Schweer T. Yarza P. (2013). The SILVA ribosomal RNA gene database project: improved data processing and web-based tools. *Nucleic Acids Res.* 41 D590–D596. 10.1093/nar/gks1219 23193283 PMC3531112

[B70] Rübsaamen E. H. (1905). Beiträge zur kenntnis aussereuropäischer zoocecidien. I, gallen von bismark archipel. *Marcellia* 4 5–25.

[B71] Russell J. A. Latorre A. Sabater-Muñoz B. Moya A. Moran N. A. (2003). Side-stepping secondary symbionts: widespread horizontal transfer across and beyond the Aphidoidea. *Mol. Ecol.* 12 1061–1075. 10.1046/j.1365-294X.2003.01780.x 12753224

[B72] Sanders J. G. Powell S. Kronauer D. J. Vasconcelos H. L. Frederickson M. E. Pierce N. E. (2014). Stability and phylogenetic correlation in gut microbiota: lessons from ants and apes. *Mol. Ecol.* 23 1268–1283. 10.1111/mec.12611 24304129

[B73] Sandström J. P. Russell J. A. White J. P. Moran N. A. (2001). Independent origins and horizontal transfer of bacterial symbionts of aphids. *Mol. Ecol.* 10 217–228. 10.1046/j.1365-294X.2001.01189.x 11251800

[B74] Scarborough C. L. Ferrari J. Godfray H. C. (2005). Aphid protected from pathogen by endosymbiont. *Science* 310:1781. 10.1126/science.1120180 16357252

[B75] Sepúlveda D. A. Zepeda-Paulo F. Ramírez C. C. Lavandero B. Figueroa C. C. (2017). Diversity, frequency, and geographic distribution of facultative bacterial endosymbionts in introduced aphid pests. *Insect Sci.* 24 511–521. 10.1111/1744-7917.12313 26773849

[B76] Simon J. C. Carre S. Boutin M. Prunier-Leterme N. Sabater-Muñoz B. Latorre A. (2003). Host-based divergence in populations of the pea aphid: insights from nuclear markers and the prevalence of facultative symbionts. *Proc. R. Soc. B Biol. Sci.* 270 1703–1712. 10.1098/rspb.2003.2430 PMC1691435 12964998

[B77] Smith A. H. Łukasik P. O’Connor M. P. Lee A. Mayo G. Drott M. T. (2015). Patterns, causes and consequences of defensive microbiome dynamics across multiple scales. *Mol. Ecol.* 24 1135–1149. 10.1111/mec.13095 25683348

[B78] Trevelline B. K. Sosa J. Hartup B. K. Kohl K. D. (2020). A bird’s-eye view of phylosymbiosis: weak signatures of phylosymbiosis among all 15 species of cranes. *Proc. R. Soc. B Biol. Sci.* 287:20192988. 10.1098/rspb.2019.2988 PMC7126031 32183630

[B79] Tsuchida T. Koga R. Fukatsu T. (2004). Host plant specialization governed by facultative symbiont. *Science* 303:1989. 10.1126/science.1094611 15044797

[B80] Tsuchida T. Koga R. Horikawa M. Tsunoda T. Maoka T. Matsumoto S. (2010). Symbiotic bacterium modifies aphid body color. *Science* 330 1102–1104. 10.1126/science.1195463 21097935

[B81] Tsuchida T. Koga R. Shibao H. Matsumoto T. Fukatsu T. (2002). Diversity and geographic distribution of secondary endosymbiotic bacteria in natural populations of the pea aphid, *Acyrthosiphon pisum*. *Mol. Ecol.* 11 2123–2135. 10.1046/j.1365-294X.2002.01606.x 12296954

[B82] Unterman B. M. Baumann P. McLean D. L. (1989). Pea aphid symbiont relationships established by analysis of 16S rRNAs. *J. Bacteriol.* 171 2970–2974. 10.1128/jb.171.6.2970-2974.1989 2470724 PMC210002

[B83] Wagner S. M. Martinez A. J. Ruan Y. Kim K. L. Lenhart P. A. Dehnel A. C. (2015). Facultative endosymbionts mediate dietary breadth in a polyphagous herbivore. *Funct. Ecol.* 29 1402–1410. 10.1111/1365-2435.12459

[B84] Wang Q. Garrity G. M. Tiedje J. M. Cole J. R. (2007). Naive Bayesian classifier for rapid assignment of rRNA sequences into the new bacterial taxonomy. *Appl. Environ. Microbiol.* 73 5261–5267. 10.1128/Aem.00062-07 17586664 PMC1950982

[B85] Wang Z. Su X. M. Wen J. Jiang L. Y. Qiao G. X. (2014). Widespread infection and diverse infection patterns of *Wolbachia* in Chinese aphids. *Insect Sci.* 21 313–325. 10.1111/1744-7917.12102 24395812

[B86] Wheeler B. Torchiano M. (2010). *Lmperm: Permutation Tests for Linear Models. R Package Version 1.1-2.* Available Online at: https://github.com/mtorchiano/lmPerm [accessed June 18, 2021].

[B87] Xu S. F. Jiang L. Y. Qiao G. X. Chen J. (2020). The bacterial flora associated with the polyphagous aphid *Aphis gossypii* Glover (Hemiptera: Aphididae) is strongly affected by host plants. *Microb. Ecol.* 79 971–984. 31802184 10.1007/s00248-019-01435-2 PMC7198476

[B88] Xu T. T. Jiang L. Y. Chen J. Qiao G. X. (2020). Host plants influence the symbiont diversity of Eriosomatinae (Hemiptera: Aphididae). *Insects* 11:217. 10.3390/insects11040217 PMC7240687 32244698

[B89] Xu T. T. Chen J. Jiang L. Y. Qiao G. X. (2018). Historical and cospeciating associations between Cerataphidini aphids (Hemiptera: Aphididae: Hormaphidinae) and their primary endosymbiont *Buchnera aphidicola*. *Zool. J. Linn. Soc.* 182 604–613. 10.1093/zoolinnean/zlx048

[B90] Xu T. T. Chen J. Jiang L. Y. Qiao G. X. (2021). Diversity of bacteria associated with Hormaphidinae aphids (Hemiptera: Aphididae). *Insect Sci*. 28 165–179. 10.1111/1744-7917.12746 31840419 PMC7818174

[B91] Zytynska S. E. Tighiouart K. Frago E. (2021). Benefits and costs of hosting facultative symbionts in plant-sucking insects: a meta-analysis. *Mol. Ecol.* 30 2483–2494. 10.1111/mec.158 33756029

