# Supplementary material for: Insights Into the Species-Specific Microbiota of Greenideinae (Hemiptera: Aphididae) With Evidence of Phylosymbiosis
Source: Front Microbiol. 2022 Feb 22;13:828170. doi: 10.3389/fmicb.2022.828170 (PMC8901875; doi:10.3389/fmicb.2022.828170)
Supplement: Supplementary file 4 [file Data_Sheet_3.PDF]

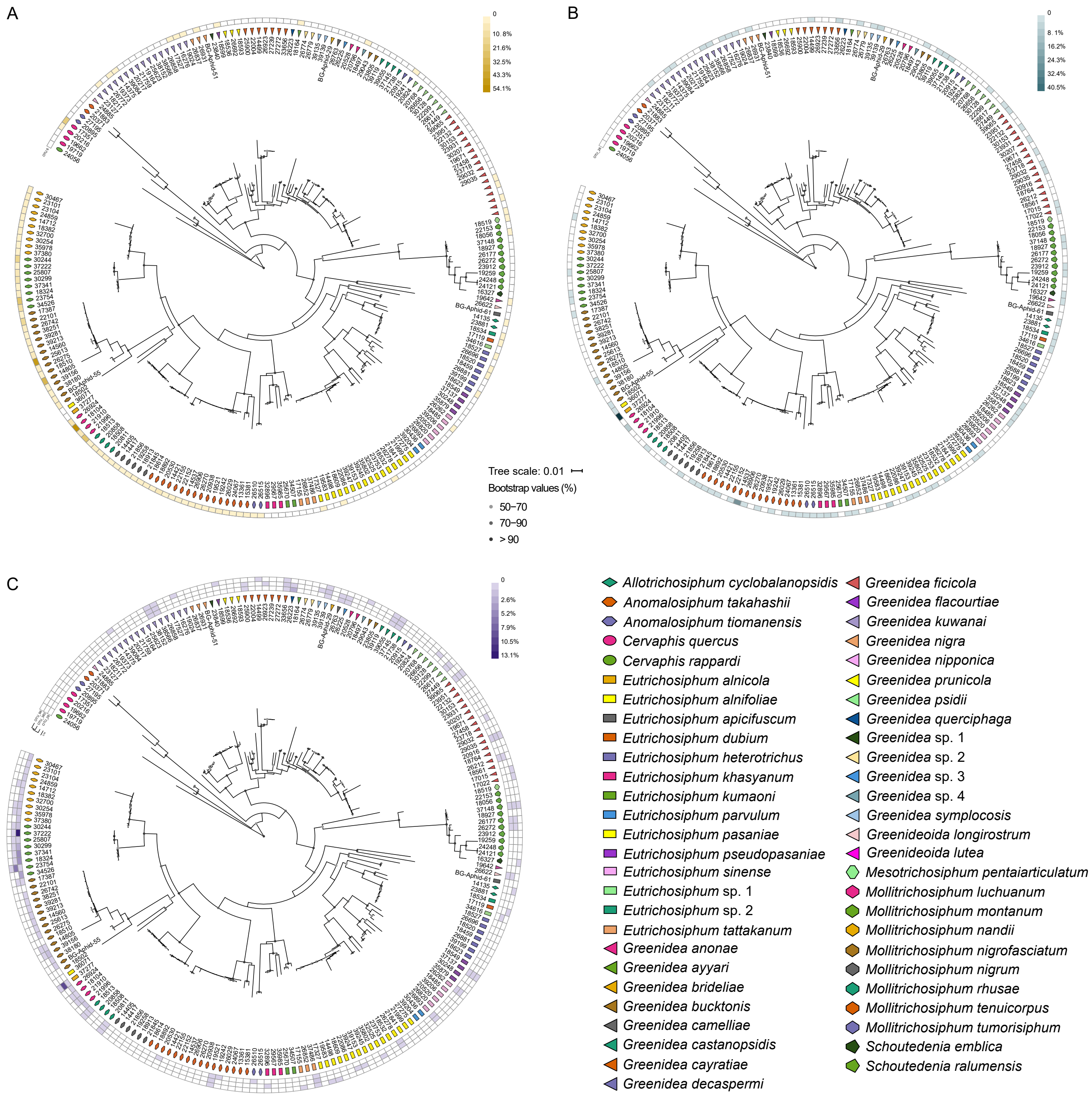

Supplementary Figure 3 Heatmap representing the distribution and relative abundances of OTUs belonging to *Rickettsia* **(A)**, *Hamiltonella defensa* **(B)** and *Fukatsuia symbiotica* **(C)**. The maximum-likelihood trees display the phylogenetic relationships of secondary symbiont OTUs and Greenideinae.
