# Supplementary material for: Insights Into the Species-Specific Microbiota of Greenideinae (Hemiptera: Aphididae) With Evidence of Phylosymbiosis
Source: Front Microbiol. 2022 Feb 22;13:828170. doi: 10.3389/fmicb.2022.828170 (PMC8901875; doi:10.3389/fmicb.2022.828170)
Supplement: Supplementary file 8 [file Data_Sheet_7.PDF]

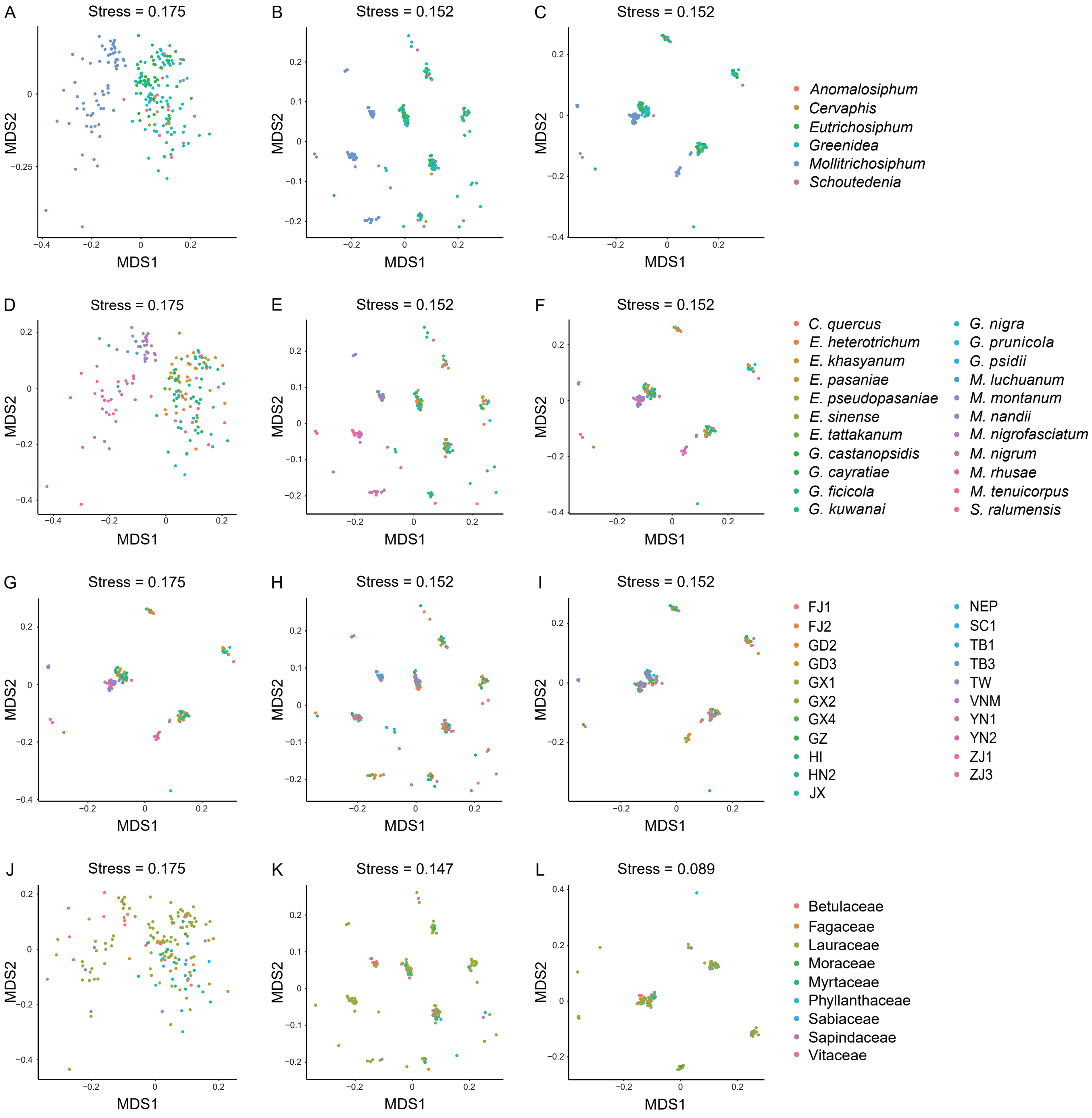

Supplementary Figure 7 Nonmetric multidimensional scaling (NMDS) plots based on unweighted UniFrac distances of bacterial **(A, D, G, J)**, symbiont **(B, E, H, K)** and secondary symbiont **(C, F, I, L)** communities (n ≥ 3). Samples are colored by aphid genus **(A–C)**, aphid species **(D–F)**, geographic region **(G–I)** and host plant **(J–L)**. The stress value indicates the goodness of fit between the NMDS representation and the data. The abbreviations are given in **Supplementary Table 3**.
