# Supplementary material for: Insights Into the Species-Specific Microbiota of Greenideinae (Hemiptera: Aphididae) With Evidence of Phylosymbiosis
Source: Front Microbiol. 2022 Feb 22;13:828170. doi: 10.3389/fmicb.2022.828170 (PMC8901875; doi:10.3389/fmicb.2022.828170)
Supplement: Supplementary file 10 [file Data_Sheet_9.PDF]

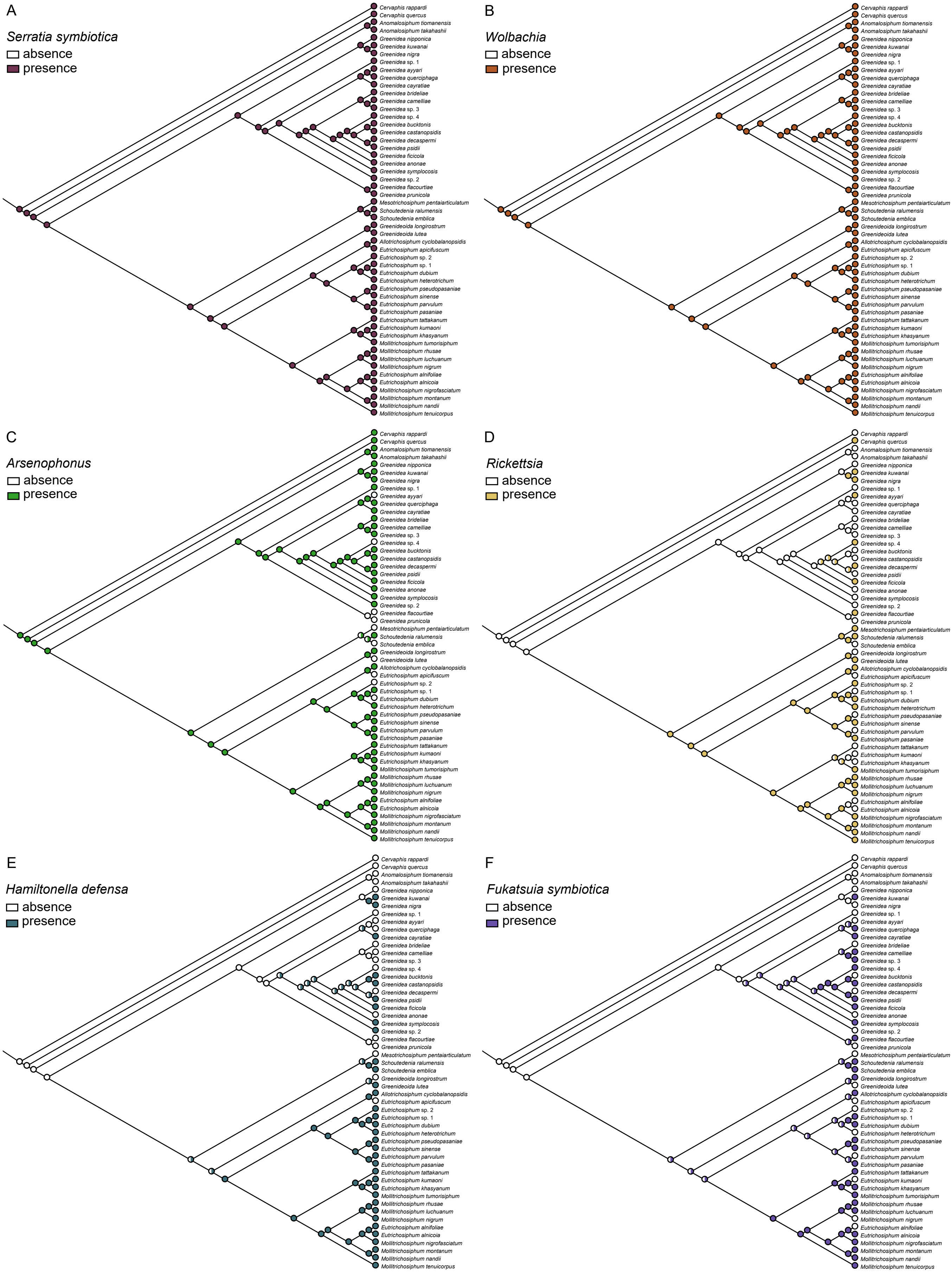

Supplementary Figure 9 Ancestral aphid-secondary symbiont associations estimated by parsimony reconstruction. The absence (white) or presence (colors) of secondary symbionts was mapped on the simplified cladogram displaying the phylogenetic relationships of Greenideinae species. Nodes that are not fully coloured refer to uncertain ancestral state.
