# Supplementary material for: Insights Into the Species-Specific Microbiota of Greenideinae (Hemiptera: Aphididae) With Evidence of Phylosymbiosis
Source: Front Microbiol. 2022 Feb 22;13:828170. doi: 10.3389/fmicb.2022.828170 (PMC8901875; doi:10.3389/fmicb.2022.828170)
Supplement: Supplementary file 11 [file Data_Sheet_10.DOCX]

**SUPPLEMENTARY METHODS**

**Maximum-Likelihood Phylogenetic Inference**

Based on the representative sequences of symbiont OTUs, the relatedness of *Buchnera* OTUs and secondary symbiont OTUs were estimated using the maximum-likelihood (ML) approach. The phylogenetic relationship of the aphid subfamily Greenideinae was evaluated using the combined dataset of cytochrome *c* oxidase subunit I (COI), cytochrome *b* (Cytb) and elongation factor-1α (EF-1α) genes. The outgroups comprised *Aiceona himalaica* (Aiceoninae), *Anoecia fulviabdominalis* (Anoeciinae), *Pineus armandicola* (Adelgidae), *Phylloxerina salicis* (Phylloxeridae), *Cinara costata* (Lachninae), *Eriosoma lanigerum* (Eriosomatinae), and six species of Aphidinae, namely, *Aphids aurantii*, *Aphids kurosawai*, *Brevicoryne brassicae*, *Hyalopterus pruni*, *Lipaphis pseudobrassicae* and *Rhopalosiphum padi*. The GenBank accession numbers of the sequences are listed in **Supplementary Table 1**. All ML analyses were performed under the GTRCAT model with 1000 rapid bootstrapping replicates in RAxML v8.2.7 (Stamatakis, 2014).

**REFERENCES**

Stamatakis, A. (2014) RAxML version 8: a tool for phylogenetic analysis and post-analysis of large phylogenies. *Bioinformatics* 30, 1312–1313. doi: 10.1093/bioinformatics/btu033

**Supplementary Table 1** Voucher information and GenBank accession numbers of aphid samples used in the present study.

| Species | Voucher | Host plant | Locality | Geo-coordinates | COI | Cytb | EF-1α |
| --- | --- | --- | --- | --- | --- | --- | --- |
| Ingroups^†^ |  |  |  |  |  |  |  |
| *Allotrichosiphum cyclobalanopsidis* | 23881 | Fagaceae | Anning, Yunnan, China | 24.98°N, 102.42°E | OL619514 |  | OL631826 |
| *Allotrichosiphum cyclobalanopsidis* | 14135 | *Quercus pachyloma* | Nantou, Taiwan, China | 23.88°N, 120.92°E | JQ926111^‡^ | JX186596^‡^ | OL631781 |
| *Anomalosiphum takahashii* | 20371 | *Dalbergia millettii* | Lishui, Zhejiang, China | 27.88°N, 119.18°E | JQ926133^‡^ | JX186598^‡^ | KF856794^‡^ |
| *Anomalosiphum takahashii* | 21883 | Fabaceae | Ruyuan, Guangdong, China | 24.09°N, 113.16°E | JQ926135^‡^ | JX186591^‡^ | OL631814 |
| *Anomalosiphum tiomanensis* | 27195 | *Phyllanthus emblica* | Nanning, Guangxi, China | 22.79°N, 108.37°E | JQ926138^‡^ | JX186594^‡^ |  |
| *Anomalosiphum tiomanensis* | 20895 | Unknown | Wenchang, Hainan, China | 19.63°N, 110.97°E | JQ926137^‡^ | JX186748^‡^ |  |
| *Cervaphis quercus* | 20216 | *Castanea mollissima* | Yuexi, Anhui, China | 31.06°N, 116.11°E | JQ926126^‡^ | OL624550 |  |
| *Cervaphis quercus* | 19662 | *Cyclobalanopsis kerrii* | Mt. Bawangling, Hainan, China | 19.03°N, 109.17°E | JQ926129^‡^ |  | KF856820^‡^ |
| *Cervaphis quercus* | 19719 | *Quercus acutissima* | Yanhe, Guizhou, China | 28.57°N, 108.51°E | JQ926127^‡^ | OL624549 | OL631806 |
| *Cervaphis quercus* | 17351 | *Quercus aliena* | Taishun, Zhejiang, China | 27.71°N, 119.68°E | JQ926130^‡^ |  | OL631789 |
| *Cervaphis rappardi* | 24056 | Unknown | Ruili, Yunnan, China | 24.06°N, 97.98°E | JQ926140^‡^ | JX186593^‡^ | KF856791^‡^ |
| *Eutrichosiphum alnicola* | 37277 | *Alnus cremastogyne* | Cona, Tibet, China | 27.87°N, 91.80°E | OL619455 | OL624622 | OL631876 |
| *Eutrichosiphum alnifoliae* | 36071 | *Alnus cremastogyne* | Huayuan, Hunan, China | 28.57°N, 109.50°E | OL619459 | OL624618 | OL631872 |
| *Eutrichosiphum apicifuscum* | BG-Aphid-61 | *Lithocarpus* sp. | Baise, Guangxi, China | 24.48°N, 106.41°E | OL619437 | OL624640 | OL631892 |
| *Eutrichosiphum dubium* | 17119 | *Quercus* sp. | Panzhihua, Sichuan, China | 27.11°N, 101.53°E | JQ926046^‡^ | JX186604^‡^ | KF856797^‡^ |
| *Eutrichosiphum heterotrichus* | 18520 | *Castanopsis fabri* | Mt. Diaoluo, Hainan, China | 18.66°N, 109.93°E | JQ926042^‡^ | JX186608^‡^ | OL631794 |
| *Eutrichosiphum heterotrichus* | 18459 | *Cyclobalanopsis patelliformis* | Mt. Jianfengling, Hainan, China | 18.71°N, 108.88°E | JQ926043^‡^ | JX186607^‡^ | KF856798^‡^ |
| *Eutrichosiphum heterotrichus* | 18527 | *Lithocarpus elmerrillii* | Mt. Diaoluo, Hainan, China | 18.66°N, 109.93°E | JQ926041^‡^ | JX186609^‡^ | OL631795 |
| *Eutrichosiphum heterotrichus* | 39199 | *Lithocarpus glaber* | Kaohsiung, Taiwan, China | 23.05°N, 120.72°E | OL619444 | OL624633 | OL631885 |
| *Eutrichosiphum heterotrichus* | 18623 | *Lithocarpus glaber* | Shixing, Guangdong, China | 24.95°N, 114.07°E | JQ926036^‡^ | JX186614^‡^ | OL631800 |
| *Eutrichosiphum heterotrichus* | 26696 | Unknown | Hangzhou, Zhejiang, China | 30.26°N, 120.12°E | OL619493 | OL624585 | OL631841 |
| *Eutrichosiphum heterotrichus* | 26881 | Unknown | Mt. Wuyi, Fujian, China | 27.63°N, 117.95°E | OL619484 | OL624594 | OL631848 |
| *Eutrichosiphum khasyanum* | 35985 | *Quercus aquifolioides* | Medog, Tibet, China | 29.87°N, 95.73°E | OL619460 | OL624617 | OL631871 |
| *Eutrichosiphum khasyanum* | 29567 | *Quercus semecarpifolia* | NyingChi, Tibet, China | 29.86°N, 95.78°E | OL619473 | OL624605 | OL631859 |
| *Eutrichosiphum khasyanum* | 32896 | *Quercus semecarpifolia* | NyingChi, Tibet, China | 29.64°N, 94.36°E | OL619466 | OL624611 | OL631866 |
| *Eutrichosiphum kumaoni* | 34507 | *Castanopsis* sp. | Dali, Yunnan, China | 25.61°N, 100.27°E | OL619464 | OL624613 | OL631799 |
| *Eutrichosiphum kumaoni* | 25670 | Fagaceae | Guilin, Guangxi, China | 25.51°N, 110.28°E | OL619506 | OL624574 |  |
| *Eutrichosiphum parvulum* | 30436 | *Trachelospermum bodinieri* | Kunming, Yunnan, China | 25.14°N, 102.74°E | OL619468 |  | OL631864 |
| *Eutrichosiphum parvulum* | 39204 | *Trachelospermum jasminoides* | Kaohsiung, Taiwan, China | 23.05°N, 120.72°E | OL619443 | OL624634 | OL631886 |
| *Eutrichosiphum pasaniae* | 22086 | *Castanopsis carlesii* | Mt. Liangye, Fujian, China | 25.03°N, 116.23°E | OL619523 | OL624558 | OL631817 |
| *Eutrichosiphum pasaniae* | 18532 | *Castanopsis fabri* | Mt. Diaoluo, Hainan, China | 18.66°N, 109.93°E | JQ926021^‡^ | JX186628^‡^ |  |
| *Eutrichosiphum pasaniae* | 19583 | *Castanopsis fabri* | Mt. Jianfengling, Hainan, China | 18.71°N, 108.88°E | JQ926014^‡^ | JX186634^‡^ | OL631805 |
| *Eutrichosiphum pasaniae* | 39245 | *Castanopsis* sp. | Pingtung, Taiwan, China | 22.24°N, 120.83°E | OL619441 | OL624636 | OL631888 |
| *Eutrichosiphum pasaniae* | 26278 | *Castanopsis* sp. | Shangsi, Guangxi, China | 22.15°N, 107.98°E | OL619498 | OL624582 | OL631837 |
| *Eutrichosiphum pasaniae* | 18609 | *Castanopsis* sp. | Shixing, Guangdong, China | 24.95°N, 114.07°E | JQ926016^‡^ | JX186632^‡^ |  |
| *Eutrichosiphum pasaniae* | 21999 | *Clerodendrum cyrtophyllum* | Shixing, Guangdong, China | 24.95°N, 114.07°E | OL619525 | OL624556 | OL631815 |
| *Eutrichosiphum pasaniae* | 23753 | Fagaceae | Jingdong, Yunnan, China | 24.27°N, 100.73°E | OL619517 | OL624564 | OL631823 |
| *Eutrichosiphum pasaniae* | 35802 | Fagaceae | Medog, Tibet, China | 29.29°N, 95.31°E | OL619462 | OL624615 | OL631869 |
| *Eutrichosiphum pasaniae* | 17327 | Fagaceae | Taishun, Zhejiang, China | 27.71°N, 119.68°E | JQ926031^‡^ | JX186619^‡^ | OL631788 |
| *Eutrichosiphum pasaniae* | 14498 | *Ilex ficoidea* | Mt. Wuyi, Fujian, China | 27.63°N, 117.95°E | JQ926035^‡^ | JX186615^‡^ | OL631783 |
| *Eutrichosiphum pasaniae* | 39153 | *Lithocarpus* sp. | Nantou, Taiwan, China | 23.88°N, 120.92°E | OL619445 | OL624632 | OL631884 |
| *Eutrichosiphum pasaniae* | 39247 | *Lithocarpus* sp. | Pingtung, Taiwan, China | 22.24°N, 120.83°E | OL619440 | OL624637 | OL631889 |
| *Eutrichosiphum pasaniae* | 32525 | *Quercus griffithii* | Mt. Wuzhi, Hainan, China | 18.90°N, 109.69°E | OL619467 | OL624610 | OL631865 |
| *Eutrichosiphum pasaniae* | 27275 | Unknown | Guilin, Guangxi, China | 25.51°N, 110.28°E | OL619479 | OL624599 | OL631854 |
| *Eutrichosiphum pasaniae* | 21841 | Unknown | Mt. Mang, Hunan, China | 24.10°N, 113.16°E | OL619526 | OL624555 | OL631813 |
| *Eutrichosiphum pseudopasaniae* | 18549 | *Castanopsis* sp. | Xinglong, Hainan, China | 18.69°N, 110.24°E | JQ926013^‡^ |  | KF856800^‡^ |
| *Eutrichosiphum pseudopasaniae* | 37137 | *Cyclobalanopsis glauca* | Nanchang, Jiangxi, China | 28.76°N, 115.83°E | OL619458 | OL624619 | OL631873 |
| *Eutrichosiphum pseudopasaniae* | 35879 | Fagaceae | Medog, Tibet, China | 29.49°N, 95.46°E | OL619461 | OL624616 | OL631870 |
| *Eutrichosiphum pseudopasaniae* | 30248 | Unknown | Dhampus, Gandakī, Nepal | 27.68°N, 85.32°E | OL619469 | OL624609 | OL631863 |
| *Eutrichosiphum sinense* | 26820 | *Castanea mollissima* | Suichang, Zhejiang, China | 28.40°N, 118.84°E | OL619488 | OL624590 |  |
| *Eutrichosiphum sinense* | 26893 | *Castanopsis eyrei* | Mt. Wuyi, Fujian, China | 27.69°N, 117.65°E | OL619483 | OL624595 | OL631849 |
| *Eutrichosiphum sinense* | 20520 | *Castanopsis ferox* | Simao, Yunnan, China | 22.75°N, 100.99°E | JQ926010^‡^ | JX186637^‡^ | KF856801^‡^ |
| *Eutrichosiphum sinense* | 39206 | *Castanopsis* sp. | Kaohsiung, Taiwan, China | 23.05°N, 120.72°E | OL619442 | OL624635 | OL631887 |
| *Eutrichosiphum sinense* | 18485 | *Castanopsis* sp. | Mt. Diaoluo, Hainan, China | 18.66°N, 109.93°E | JQ926011^‡^ | JX186636^‡^ |  |
| *Eutrichosiphum sinense* | 26262 | *Castanopsis* sp. | Shangsi, Guangxi, China | 22.15°N, 107.98°E | OL619500 | OL624580 | OL631836 |
| *Eutrichosiphum* sp. 1 | 34616 | *Cyclobalanopsis glauca* | Gongshan, Yunnan, China | 27.63°N, 98.63°E | OL619463 | OL624614 | OL631868 |
| *Eutrichosiphum* sp. 2 | 18534 | *Castanopsis* sp. | Mt. Diaoluo, Hainan, China | 18.66°N, 109.93°E | JQ926047^‡^ | JX186603^‡^ | KF856796^‡^ |
| *Eutrichosiphum tattakanum* | 37486 | *Quercus semecarpifolia* | Zhouzhi, Shaanxi, China | 33.82°N, 107.74°E | OL619454 | OL624623 | OL631877 |
| *Eutrichosiphum tattakanum* | 17155 | *Quercus* sp. | Panzhihua, Sichuan, China | 27.04°N, 101.98°E | JQ926007^‡^ | OL624545 | OL631787 |
| *Eutrichosiphum tattakanum* | 26852 | *Quercus* sp. | Qingyuan, Zhejiang, China | 27.83°N, 119.22°E | OL619486 | OL624592 | OL631846 |
| *Greenidea anonae* | 20528 | Fagaceae | Simao, Yunnan, China | 22.75°N, 100.99°E | JQ926001^‡^ | JX186641^‡^ | OL631807 |
| *Greenidea anonae* | 20796 | *Glochidion puberum* | Mt. Yinggeling, Hainan, China | 19.37°N, 109.46°E | JQ925999^‡^ | JX186643^‡^ | OL631809 |
| *Greenidea ayyari* | 18164 | *Cyclobalanopsis glauca* | Kunming, Yunnan, China | 25.06°N, 102.76°E | JQ925998^‡^ | JX186644^‡^ | KF856803^‡^ |
| *Greenidea brideliae* | BG-Aphid-29 | *Bridelia tomentosa* | Chiayi, Taiwan, China | 23.48°N, 120.46°E | OL619439 | OL624638 | OL631890 |
| *Greenidea bucktonis* | 23805 | *Duabanga grandiflora* | Yun, Yunnan, China | 24.10°N, 99.86°E | OL619516 | OL624565 | OL631824 |
| *Greenidea bucktonis* | 29043 | *Psidium guajava* | Quan Ba, Ha Giang, Vietnam | 12.16°N, 108.06°E | OL619474 | OL624604 | OL631893 |
| *Greenidea camelliae* | 26763 | Theaceae | Mt. West Tianmu, Zhejiang, China | 30.32°N, 119.44°E | OL619492 | OL624586 | OL631842 |
| *Greenidea castanopsidis* | 39119 | *Bridelia* sp. | Nantou, Taiwan, China | 23.88°N, 120.92°E | OL619448 | OL624629 | OL631881 |
| *Greenidea castanopsidis* | 39055 | *Bridelia* sp. | Taipei, Taiwan, China | 24.83°N, 121.52°E | OL619451 | OL624626 | OL631894 |
| *Greenidea castanopsidis* | 37145 | *Buxus sinica* | Nanchang, Jiangxi, China | 28.76°N, 115.83°E | OL619457 | OL624620 | OL631874 |
| *Greenidea castanopsidis* | 21738 | Rutaceae | Liling, Hunan, China | 26.05°N, 114.04°E | OL619528 | OL624553 |  |
| *Greenidea cayratiae* | 26923 | *Ampelopsis glandulosa* | Sanming, Fujian, China | 26.44°N, 117.59°E | OL619482 | OL624596 | OL631850 |
| *Greenidea cayratiae* | 27239 | *Ampelopsis* sp. | Guilin, Guangxi, China | 25.51°N, 110.28°E | OL619480 | OL624598 | OL631852 |
| *Greenidea cayratiae* | 27272 | *Cardiospermum halicacabum* | Guilin, Guangxi, China | 25.51°N, 110.28°E | JQ926134^‡^ | JX186595^‡^ | OL631853 |
| *Greenidea cayratiae* | 33656 | Unknown | Mt. Fanjing, Guizhou, China | 27.84°N, 108.77°E | OL619465 | OL624612 | OL631867 |
| *Greenidea cayratiae* | 22004 | Unknown | Shixing, Guangdong, China | 24.95°N, 114.07°E | OL619524 | OL624557 | OL631816 |
| *Greenidea cayratiae* | 14499 | Vitaceae | Mt. Wuyi, Fujian, China | 27.63°N, 117.95°E | JQ925994^‡^ | JX186647^‡^ | KF856807^‡^ |
| *Greenidea cayratiae* | 25900 | *Vitis* sp. | Gyirong, Tibet, China | 28.40°N, 85.35°E | OL619505 | OL624575 | OL631832 |
| *Greenidea decaspermi* | 19241 | *Litchi chinensis* | Huizhou, Guangdong, China | 23.10°N, 114.42°E | JQ925991^‡^ | OL624548 | OL631803 |
| *Greenidea decaspermi* | 20915 | *Litchi chinensis* | Xinglong, Hainan, China | 18.74°N, 110.18°E | OL619530 | OL624551 | OL631810 |
| *Greenidea ficicola* | 27458 | *Annona squamosa* | Mt. Limu, Hainan, China | 18.89°N, 110.03°E | OL619477 | OL624601 | OL631856 |
| *Greenidea ficicola* | 29032 | *Ficus elastica* | Quan Ba, Ha Giang, Vietnam | 12.16°N, 108.06°E | OL619476 | OL624602 | OL631857 |
| *Greenidea ficicola* | 30207 | *Ficus elastica* | Shenzhen, Guangdong, China | 22.58°N, 114.17°E | OL619470 | OL624608 | OL631862 |
| *Greenidea ficicola* | 39065 | *Ficus fistulosa* | Taipei, Taiwan, China | 25.03°N, 121.56°E | OL619450 | OL624627 | OL631879 |
| *Greenidea ficicola* | 19671 | *Ficus hispida* | Danzhou, Hainan, China | 19.51°N, 109.50°E | JQ925981^‡^ | JX186655^‡^ | OL631896 |
| *Greenidea ficicola* | 20916 | *Ficus hispida* | Xinglong, Hainan, China | 18.74°N, 110.18°E | OL619529 | OL624552 | OL631811 |
| *Greenidea ficicola* | 23931 | *Ficus maclellandi* | Jinghong, Yunnan, China | 21.93°N, 101.25°E | OL619512 | OL624568 | OL631827 |
| *Greenidea ficicola* | 18764 | *Ficus microcarpa* | Dali, Yunnan, China | 25.58°N, 100.23°E | JQ925984^‡^ | OL624547 |  |
| *Greenidea ficicola* | 23718 | *Ficus microcarpa* | Jingdong, Yunnan, China | 24.33°N, 100.83°E | OL619518 | OL624563 | OL631822 |
| *Greenidea ficicola* | 23951 | *Ficus microcarpa* | Jinghong, Yunnan, China | 21.93°N, 101.25°E | OL619511 | OL624569 | OL631828 |
| *Greenidea ficicola* | 22132 | *Ficus microcarpa* | Longyan, Fujian, China | 25.03°N, 116.23°E | OL619522 | OL624559 | OL631818 |
| *Greenidea ficicola* | 26212 | *Ficus microcarpa* | Napo, Guangxi, China | 23.39°N, 105.83°E | OL619503 | OL624577 | OL631833 |
| *Greenidea ficicola* | 30153 | *Ficus microcarpa* | Zhanjiang, Guangdong, China | 21.57°N, 109.76°E | OL619471 | OL624607 | OL631861 |
| *Greenidea ficicola* | 18561 | *Ficus* sp. | Guangzhou, Guangdong, China | 23.14°N, 113.26°E | JQ925986^‡^ | JX186653^‡^ | OL631797 |
| *Greenidea ficicola* | 17022 | *Ficus* sp. | Panzhihua, Sichuan, China | 26.58°N, 101.72°E | JQ925988^‡^ | JX186651^‡^ | OL631786 |
| *Greenidea ficicola* | 29035 | *Ficus* sp. | Quan Ba, Ha Giang, Vietnam | 12.16°N, 108.06°E | OL619475 | OL624603 | OL631858 |
| *Greenidea ficicola* | 17015 | Oleaceae | Dechang, Sichuan, China | 27.40°N, 102.18°E | JQ925989^‡^ | JX186650^‡^ | OL631785 |
| *Greenidea flacourtiae* | 18599 | *Castanopsis* sp. | Ruyuan, Guangdong, China | 24.75°N, 112.86°E | JQ925973^‡^ | JX186661^‡^ | KF856810^‡^ |
| *Greenidea kuwanai* | 19373 | *Castanea mollissima* | Taibai, Shaanxi, China | 33.83°N, 110.05°E | JQ925955^‡^ | JX186676^‡^ | OL631804 |
| *Greenidea kuwanai* | 18211 | *Cerasus pseudocerasus* | Chuxiong, Yunnan, China | 25.16°N, 102.06°E | JQ925960^‡^ | JX186671^‡^ | OL631792 |
| *Greenidea kuwanai* | 16276 | *Cyclobalanopsis glauca* | Mt. Leigong, Guizhou, China | 26.43°N, 108.27°E | JQ925964^‡^ | JX186667^‡^ | OL631784 |
| *Greenidea kuwanai* | 38566 | *Cyclobalanopsis glauca* | Nanjiang, Sichuan, China | 32.62°N, 106.83°E | OL619452 | OL624625 | OL631897 |
| *Greenidea kuwanai* | 25623 | *Cyclobalanopsis glauca* | Suiyang, Guizhou, China | 28.25°N, 107.45°E | OL619507 | OL624573 | OL631831 |
| *Greenidea kuwanai* | 23127 | *Cyclobalanopsis glauca* | Xichang, Sichuan, China | 27.88°N, 102.26°E | OL619519 | OL624562 | OL631821 |
| *Greenidea kuwanai* | 21759 | Fagaceae | Guidong, Hunan, China | 25.10°N, 113.07°E | OL619527 | OL624554 | OL631812 |
| *Greenidea kuwanai* | 26772 | *Quercus acutissima* | Mt. West Tianmu, Zhejiang, China | 30.34°N, 119.40°E | OL619491 | OL624587 |  |
| *Greenidea kuwanai* | 38152 | *Quercus fabri* | Ningbo, Zhejiang, China | 29.72°N, 121.07°E | OL619453 | OL624624 | OL631878 |
| *Greenidea kuwanai* | 17527 | *Quercus mongolica* | Anshan, Liaoning, China | 41.02°N, 123.10°E | JQ925961^‡^ | JX186670^‡^ | OL631790 |
| *Greenidea kuwanai* | 19164 | *Quercus mongolica* | Beijing, China | 39.97°N, 115.44°E | JQ925956^‡^ | JX186675^‡^ | OL631802 |
| *Greenidea kuwanai* | 20217 | *Quercus serrata* | Yuexi, Anhui, China | 31.06°N, 116.11°E | JQ925951^‡^ | JX186680^‡^ | KF856811^‡^ |
| *Greenidea kuwanai* | 14375 | *Quercus* sp. | Dalian, Liaoning, China | 39.53°N, 123.00°E | JQ925970^‡^ | OL624544 | OL631782 |
| *Greenidea kuwanai* | 19024 | *Quercus* sp. | Guilin, Guangxi, China | 26.04°N, 110.71°E | JQ925958^‡^ | JX186673^‡^ | OL631801 |
| *Greenidea kuwanai* | 26858 | *Quercus* sp. | Qingyuan, Zhejiang, China | 27.83°N, 119.22°E | OL619485 | OL624593 | OL631847 |
| *Greenidea kuwanai* | 39084 | *Quercus variabilis* | Nantou, Taiwan, China | 23.88°N, 120.92°E | OL619449 | OL624628 | OL631880 |
| *Greenidea nigra* | 26837 | *Cyclobalanopsis phanera* | Mt. Fengyang, Zhejiang, China | 27.89°N, 119.17°E | OL619487 | OL624591 | OL631845 |
| *Greenidea nigra* | 26931 | Fagaceae | Longyan, Fujian, China | 25.35°N, 116.83°E | OL619481 | OL624597 | OL631851 |
| *Greenidea nigra* | BG-Aphid-51 | *Lithocarpus glaber* | Tainan, Taiwan, China | 23.27°N, 120.50°E | OL619438 | OL624639 | OL631891 |
| *Greenidea nipponica* | 24885 | *Quercus* sp. | Kunming, Yunnan, China | 25.09°N, 102.78°E | OL619508 | OL624572 | OL631830 |
| *Greenidea prunicola* | 18593 | *Castanopsis carlesii* | Ruyuan, Guangdong, China | 24.09°N, 113.16°E | JQ925943^‡^ | JX186688^‡^ | OL631798 |
| *Greenidea prunicola* | 18536 | *Castanopsis fabri* | Mt. Diaoluo, Hainan, China | 18.66°N, 109.93°E | JQ925945^‡^ | JX186686^‡^ | OL631796 |
| *Greenidea prunicola* | 26692 | Fagaceae | Hangzhou, Zhejiang, China | 30.26°N, 120.12°E | OL619494 |  |  |
| *Greenidea psidii* | 26617 | *Psidium guajava* | Baoting, Hainan, China | 18.68°N, 109.57°E | OL619497 | OL624583 | OL631838 |
| *Greenidea psidii* | 30128 | *Psidium guajava* | Beihai, Guangxi, China | 21.06°N, 109.14°E | OL619472 | OL624606 | OL631860 |
| *Greenidea psidii* | 26656 | *Psidium guajava* | Danzhou, Hainan, China | 19.45°N, 109.67°E | OL619495 | OL624584 | OL631840 |
| *Greenidea psidii* | 27449 | *Psidium guajava* | Mt. Diaoluo, Hainan, China | 18.66°N, 109.93°E | OL619478 | OL624600 | OL631855 |
| *Greenidea psidii* | 20768 | *Psidium guajava* | Mt. Limu, Hainan, China | 18.89°N, 110.03°E | JQ925940^‡^ | JX186691^‡^ | OL631808 |
| *Greenidea psidii* | 20824 | *Psidium guajava* | Mt. Wuzhi, Hainan, China | 18.90°N, 109.69°E | JQ925937^‡^ | JX186693^‡^ | KF856814^‡^ |
| *Greenidea psidii* | 22299 | *Psidium guajava* | Wenchang, Hainan, China | 19.06°N, 110.08°E | OL619520 | OL624561 | OL631820 |
| *Greenidea querciphaga* | 26223 | Unknown | Napo, Guangxi, China | 23.07°N, 105.93°E | OL619502 | OL624578 | OL631834 |
| *Greenidea* sp. 1 | 23840 | *Machilus nanmu* | Cangyuan, Yunnan, China | 23.29°N, 99.10°E | OL619515 | OL624566 | OL631825 |
| *Greenidea* sp. 2 | 26779 | *Broussonetia kazinoki* | Mt. West Tianmu, Zhejiang, China | 30.34°N, 119.40°E | OL619489 | OL624589 | OL631844 |
| *Greenidea* sp. 2 | 26774 | *Hovenia acerba* | Mt. West Tianmu, Zhejiang, China | 30.34°N, 119.40°E | OL619490 | OL624588 | OL631843 |
| *Greenidea* sp. 3 | 26225 | Unknown | Napo, Guangxi, China | 23.07°N, 105.93°E | OL619501 | OL624579 | OL631835 |
| *Greenidea* sp. 4 | 18497 | *Sarcosperma laurinum* | Mt. Diaoluo, Hainan, China | 18.66°N, 109.93°E | JQ926141^‡^ | JX186694^‡^ | KF856806^‡^ |
| *Greenidea symplocosis* | 39139 | *Engelhardia roxburghiana* | Nantou, Taiwan, China | 23.88°N, 120.92°E | OL619446 | OL624631 | OL631883 |
| *Greenidea symplocosis* | 39135 | *Myrica rubra* | Nantou, Taiwan, China | 23.88°N, 120.92°E | OL619447 | OL624630 | OL631882 |
| *Greenideoida longirostrum* | 26622 | *Litsea* sp. | Mt. Wuzhi, Hainan, China | 18.90°N, 109.69°E | OL619496 |  | OL631839 |
| *Greenideoida lutea* | 19642 | *Litsea cubeba* | Mt. Bawangling, Hainan, China | 19.09°N, 109.20°E | JQ926050^‡^ | JX186739^‡^ | KF856816^‡^ |
| *Mesotrichosiphum pentaiarticulatum* | 18519 | *Castanopsis* sp. | Mt. Diaoluo, Hainan, China | 18.66°N, 109.93°E | JQ926002^‡^ | JX186698^‡^ | OL631793 |
| *Mollitrichosiphum luchuanum* | 18104 | *Meliosma rigida* | Mt. Wuyi, Fujian, China | 27.63°N, 117.95°E | JQ926105^‡^ | JF969368^‡^ | JN645024^‡^ |
| *Mollitrichosiphum luchuanum* | 26924 | Unknown | Sanming, Fujian, China | 24.07°N, 114.21°E | MT556456^‡^ | MT563140^‡^ | MT563128^‡^ |
| *Mollitrichosiphum luchuanum* | 21910 | Unknown | Shixing, Guangdong, China | 24.95°N, 114.07°E | JQ926106^‡^ | JF969389^‡^ | JX255392^‡^ |
| *Mollitrichosiphum luchuanum* | 21996 | Unknown | Shixing, Guangdong, China | 24.95°N, 114.07°E | MT556450^‡^ | MT563136^‡^ |  |
| *Mollitrichosiphum montanum* | 37222 | *Alnus cremastogyne* | Cona, Tibet, China | 27.87°N, 91.80°E | MT556464^‡^ | MT563148^‡^ | MT563131^‡^ |
| *Mollitrichosiphum montanum* | 34526 | *Alnus cremastogyne* | Dali, Yunnan, China | 25.70°N, 100.13°E | MT556462^‡^ | MT563146^‡^ | MT563130^‡^ |
| *Mollitrichosiphum montanum* | 37341 | *Alnus cremastogyne* | Medog, Tibet, China | 29.68°N, 95.50°E | MT556465^‡^ | MT563149^‡^ | MT563132^‡^ |
| *Mollitrichosiphum montanum* | 23754 | *Alnus nepalensis* | Qingliang, Yunnan, China | 24.33°N, 100.83°E | JQ926102^‡^ | JF969387^‡^ | JN645040^‡^ |
| *Mollitrichosiphum montanum* | 25807 | Fagaceae | Zhangmu, Tibet, China | 27.99°N, 85.98°E | JN644998^‡^ | JN645014^‡^ | JN645053^‡^ |
| *Mollitrichosiphum montanum* | 30299 | Unknown | Nagarkot, Bagmatī, Nepal | 27.74°N, 85.38°E | MT556459^‡^ | MT563143^‡^ |  |
| *Mollitrichosiphum montanum* | 30244 | Unknown | Dhampus, Gandakī, Nepal | 27.68°N, 85.32°E | MT556457^‡^ | MT563141^‡^ | MT563129^‡^ |
| *Mollitrichosiphum montanum* | 18324 | Unknown | NyingChi, Tibet, China | 28.72°N, 96.78°E | JQ926103^‡^ | JF969393^‡^ | JN645018^‡^ |
| *Mollitrichosiphum nandii* | 14712 | *Alnus cremastogyne* | Baoshan, Yunnan, China | 25.11°N, 99.28°E | JQ926101^‡^ | JF969364^‡^ | JQ418318^‡^ |
| *Mollitrichosiphum nandii* | 24859 | *Alnus cremastogyne* | Chuxiong, Yunnan, China | 25.00°N, 101.46°E | MT556452^‡^ | MT563138^‡^ |  |
| *Mollitrichosiphum nandii* | 35978 | *Alnus cremastogyne* | Medog, Tibet, China | 29.29°N, 95.31°E | MT556463^‡^ | MT563147^‡^ | MT563156^‡^ |
| *Mollitrichosiphum nandii* | 37380 | *Alnus cremastogyne* | Medog, Tibet, China | 29.29°N, 95.31°E | MT556466^‡^ | MT563150^‡^ | MT563133^‡^ |
| *Mollitrichosiphum nandii* | 18382 | *Fagus longipetiolata* | Tangmai, Tibet, China | 30.10°N, 95.07°E | JQ926099^‡^ | JF969369^‡^ | JQ418323^‡^ |
| *Mollitrichosiphum nandii* | 30254 | Unknown | Dhampus, Gandakī, Nepal | 27.68°N, 85.32°E | MT556458^‡^ | MT563142^‡^ | MT563158^‡^ |
| *Mollitrichosiphum nandii* | 30467 | Unknown | Mt. Huanglian, Yunnan, China | 22.89°N, 102.30°E | MT556460^‡^ | MT563144^‡^ |  |
| *Mollitrichosiphum nandii* | 32700 | Unknown | Nyalam, Tibet, China | 27.99°N, 85.98°E | MT556461^‡^ | MT563145^‡^ | MT563157^‡^ |
| *Mollitrichosiphum nandii* | 23101 | Unknown | Xichang, Sichuan, China | 27.88°N, 102.26°E | JQ926148^‡^ | JF969394^‡^ | JX255393^‡^ |
| *Mollitrichosiphum nandii* | 23104 | Unknown | Xichang, Sichuan, China | 27.88°N, 102.26°E | MT556451^‡^ | MT563137^‡^ |  |
| *Mollitrichosiphum nigrofasciatum* | 18502 | *Castanopsis fabri* | Lingshui, Hainan, China | 18.66°N, 109.93°E | JQ926091^‡^ | JN645008^‡^ | JQ418326^‡^ |
| *Mollitrichosiphum nigrofasciatum* | 26742 | *Cyclobalanopsis glauca* | Anji, Zhejiang, China | 30.41°N, 119.42°E | MT556454^‡^ |  | JQ418338^‡^ |
| *Mollitrichosiphum nigrofasciatum* | 39281 | *Cyclobalanopsis glauca* | Hualien, Taiwan, China | 24.19°N, 121.49°E | MT556471^‡^ |  |  |
| *Mollitrichosiphum nigrofasciatum* | 38251 | *Cyclobalanopsis glauca* | Lishui, Zhejiang, China | 27.91°N, 119.67°E | MT556468^‡^ |  | MT563154^‡^ |
| *Mollitrichosiphum nigrofasciatum* | 14805 | *Cyclobalanopsis glauca* | Mt. Wuyi, Fujian, China | 27.79°N, 117.79°E | JQ926097^‡^ | JF969395^‡^ | JQ418319^‡^ |
| *Mollitrichosiphum nigrofasciatum* | 39156 | *Cyclobalanopsis glauca* | Nantou, Taiwan, China | 23.88°N, 120.92°E | MT556469^‡^ |  | MT563134^‡^ |
| *Mollitrichosiphum nigrofasciatum* | 39213 | *Cyclobalanopsis glauca* | Pingtung, Taiwan, China | 22.24°N, 120.83°E | MT556470^‡^ |  | MT563153^‡^ |
| *Mollitrichosiphum nigrofasciatum* | BG-Aphid-55 | *Cyclobalanopsis glauca* | Taipei, Taiwan, China | 25.01°N, 121.54°E | MT556472^‡^ | MT563152^‡^ | MT563135^‡^ |
| *Mollitrichosiphum nigrofasciatum* | 38180 | *Cyclobalanopsis* sp. | Zhoushan, Zhejiang, China | 29.84°N, 122.24°E | MT556467^‡^ | MT563151^‡^ | MT563155^‡^ |
| *Mollitrichosiphum nigrofasciatum* | 18510 | *Lithocarpus elmerrillii* | Lingshui, Hainan, China | 18.66°N, 109.93°E | JQ926090^‡^ | JN645010^‡^ | JQ418328^‡^ |
| *Mollitrichosiphum nigrofasciatum* | 22101 | *Lithocarpus glaber* | Mt. Liangye, Fujian, China | 25.29°N, 116.28°E | JQ926085^‡^ | JF969400^‡^ | JN645036^‡^ |
| *Mollitrichosiphum nigrofasciatum* | 14560 | *Lithocarpus glaber* | Mt. Wuyi, Fujian, China | 27.79°N, 117.79°E | JQ926098^‡^ | JF969363^‡^ | JN645022^‡^ |
| *Mollitrichosiphum nigrofasciatum* | 17387 | *Quercus aliena* | Taishun, Zhejiang, China | 27.71°N, 119.68°E | JQ926093^‡^ |  | MT563127^‡^ |
| *Mollitrichosiphum nigrofasciatum* | 26275 | Unknown | Mt. Shiwandashan, Guangxi, China | 21.91°N, 107.9°E | JN645001^‡^ | JN645017^‡^ | JN645051^‡^ |
| *Mollitrichosiphum nigrofasciatum* | 25613 | Unknown | Zunyi, Guizhou, China | 28.25°N, 107.45°E | MT556453^‡^ |  |  |
| *Mollitrichosiphum nigrum* | 19258 | *Ailanthus altissima* | Xing’an, Guangxi, China | 25.62°N, 110.65°E | JQ926081^‡^ | JF969377^‡^ | JN645027^‡^ |
| *Mollitrichosiphum nigrum* | 14405 | *Castanea* sp. | Mt. Wuyi, Fujian, China | 27.63°N, 117.95°E | JQ926083^‡^ | JN645004^‡^ | JN645019^‡^ |
| *Mollitrichosiphum nigrum* | 14417 | *Elaeagnus pungens* | Mt. Wuyi, Fujian, China | 27.63°N, 117.95°E | JQ926084^‡^ | JF969359^‡^ | JN645052^‡^ |
| *Mollitrichosiphum nigrum* | 18913 | *Meliosma cuneifolia* | Longsheng, Guangxi, China | 25.80°N, 110.02°E | JQ926082^‡^ | JF969375^‡^ | JN645026^‡^ |
| *Mollitrichosiphum nigrum* | 21845 | Unknown | Mt. Mang, Hunan, China | 24.10°N, 113.16°E | JQ926080^‡^ | JF969390^‡^ | JN645032^‡^ |
| *Mollitrichosiphum nigrum* | 21856 | Unknown | Ruyuan, Guangdong, China | 24.09°N, 113.16°E | JQ926079^‡^ | JF969391^‡^ | JN645033^‡^ |
| *Mollitrichosiphum rhusae* | 18513 | Fagaceae | Mt. Diaoluo, Hainan, China | 18.66°N, 109.93°E | JQ926075^‡^ | JF969373^‡^ | JQ418329^‡^ |
| *Mollitrichosiphum rhusae* | 20811 | Fagaceae | Mt. Wuzhi, Hainan, China | 18.90°N, 109.69°E | JQ926073^‡^ | JF969380^‡^ | JQ418330^‡^ |
| *Mollitrichosiphum rhusae* | 18508 | *Helicia hainanensis* | Mt. Diaoluo, Hainan, China | 18.66°N, 109.93°E | JQ926077^‡^ | JF969371^‡^ | JX255395^‡^ |
| *Mollitrichosiphum rhusae* | 20858 | Meliaceae | Mt. Diaoluo, Hainan, China | 18.66°N, 109.93°E | JQ926072^‡^ | JF969381^‡^ | JN645029^‡^ |
| *Mollitrichosiphum tenuicorpus* | 15381 | *Alnus cremastogyne* | Medog, Tibet, China | 29.29°N, 95.31°E | JQ926068^‡^ | JF969366^‡^ | JN645044^‡^ |
| *Mollitrichosiphum tenuicorpus* | 14421 | *Castanea* sp. | Mt. Wuyi, Fujian, China | 27.63°N, 117.95°E | JQ926070^‡^ | JF969360^‡^ | JN645042^‡^ |
| *Mollitrichosiphum tenuicorpus* | 24067 | *Castanopsis calathiformis* | Ruili, Yunnan, China | 24.07°N, 97.82°E | JF969343^‡^ | JF969392^‡^ | JQ418331^‡^ |
| *Mollitrichosiphum tenuicorpus* | 18614 | *Castanopsis carlesii* | Shixing, Guangdong, China | 24.95°N, 114.07°E | JQ926066^‡^ | JF969396^‡^ | JN645049^‡^ |
| *Mollitrichosiphum tenuicorpus* | 26029 | *Castanopsis eyrei* | Huaping, Guangxi, China | 25.06°N, 109.09°E | JN644999^‡^ | JN645015^‡^ | JN645054^‡^ |
| *Mollitrichosiphum tenuicorpus* | 20938 | *Castanopsis fabri* | Mt. Jianfengling, Hainan, China | 18.71°N, 108.88°E | JQ926060^‡^ | JF969383^‡^ | JN645046^‡^ |
| *Mollitrichosiphum tenuicorpus* | 20530 | *Castanopsis ferox* | Simao, Yunnan, China | 22.75°N, 100.99°E | JQ926062^‡^ | JF969379^‡^ | JN645028^‡^ |
| *Mollitrichosiphum tenuicorpus* | 14537 | *Castanopsis sclerophylla* | Mt. Wuyi, Fujian, China | 27.63°N, 117.95°E | JQ926069^‡^ | JF969362^‡^ | JN645043^‡^ |
| *Mollitrichosiphum tenuicorpus* | 19242 | Fagaceae | Changjiang, Hainan, China | 19.09°N, 109.20°E | JQ926064^‡^ | JF969376^‡^ | JN645045^‡^ |
| *Mollitrichosiphum tenuicorpus* | 18892 | Fagaceae | Guilin, Guangxi, China | 25.06°N, 109.09°E | JQ926065^‡^ | JF969397^‡^ | JN645025^‡^ |
| *Mollitrichosiphum tenuicorpus* | 26906 | Fagaceae | Jiangle, Fujian, China | 26.51°N, 117.29°E | MT556455^‡^ | MT563139^‡^ | JQ418340^‡^ |
| *Mollitrichosiphum tenuicorpus* | 26270 | Fagaceae | Shangsi, Guangxi, China | 22.15°N, 107.98°E | JQ418313^‡^ | JQ418317^‡^ | JQ418334^‡^ |
| *Mollitrichosiphum tenuicorpus* | 13361 | *Meliosma rigida* | Baoshan, Yunnan, China | 25.29°N, 98.81°E | JQ926071^‡^ | JF969357^‡^ | JN645020^‡^ |
| *Mollitrichosiphum tenuicorpus* | 19521 | *Quercus* sp. | Mt. Jianfengling, Hainan, China | 18.71°N, 108.88°E | JQ926063^‡^ | JF969378^‡^ | JN645048^‡^ |
| *Mollitrichosiphum tenuicorpus* | 22152 | Unknown | Nanjing, Fujian, China | 24.05°N, 117.27°E | JQ926059^‡^ | JF969384^‡^ | JN645037^‡^ |
| *Mollitrichosiphum tenuicorpus* | 22155 | Unknown | Zhangzhou, Fujian, China | 24.05°N, 117.27°E | JQ926058^‡^ | JF969385^‡^ | JN645038^‡^ |
| *Mollitrichosiphum tumorisiphum* | 26515 | Fagaceae | Hualien, Taiwan, China | 24.19°N, 121.49°E | JN645003^‡^ | JQ418316^‡^ | JQ418336^‡^ |
| *Mollitrichosiphum tumorisiphum* | 26510 | Fagaceae | Mt. Taman, Taiwan, China | 24.71°N, 121.45°E | JN645002^‡^ | JQ418315^‡^ | JQ418335^‡^ |
| *Schoutedenia emblica* | 16327 | Thelypteridaceae | Lushui, Yunnan, China | 25.85°N, 98.85°E | JQ926125^‡^ | JX186740^‡^ | KF856817^‡^ |
| *Schoutedenia ralumensis* | 24248 | *Breynia fruticosa* | Mt. Jianfengling, Hainan, China | 18.71°N, 108.88°E | OL619509 | OL624571 | OL631895 |
| *Schoutedenia ralumensis* | 24121 | *Breynia fruticosa* | Mt. Yinggeling, Hainan, China | 19.37°N, 109.46°E | OL619510 | OL624570 | OL631829 |
| *Schoutedenia ralumensis* | 19259 | *Flueggea* sp. | Guilin, Guangxi, China | 25.32°N, 110.39°E | JQ926119^‡^ | JX186745^‡^ |  |
| *Schoutedenia ralumensis* | 18927 | *Glochidion puberum* | Guilin, Guangxi, China | 25.08°N, 109.05°E | JQ926121^‡^ | JX186743^‡^ |  |
| *Schoutedenia ralumensis* | 37148 | *Glochidion puberum* | Taihe, Jiangxi, China | 26.58°N, 115.18°E | OL619456 | OL624621 | OL631875 |
| *Schoutedenia ralumensis* | 18056 | *Glochidion wilsonii* | Mt. Wuyi, Fujian, China | 27.63°N, 117.95°E | JQ926112^‡^ | OL624546 | OL631791 |
| *Schoutedenia ralumensis* | 26177 | Unknown | Daxin, Guangxi, China | 22.91°N, 106.76°E | OL619504 | OL624576 |  |
| *Schoutedenia ralumensis* | 23912 | Unknown | Jinghong, Yunnan, China | 22.00°N, 100.76°E | OL619513 | OL624567 | OL631898 |
| *Schoutedenia ralumensis* | 26272 | Unknown | Shangsi, Guangxi, China | 22.15°N, 107.98°E | OL619499 | OL624581 |  |
| *Schoutedenia ralumensis* | 22153 | Unknown | Zhangzhou, Fujian, China | 24.05°N, 117.27°E | OL619521 | OL624560 | OL631819 |
| Outgroups^†^ |  |  |  |  |  |  |  |
| *Anoecia fulviabdominalis* |  |  |  |  | KM501362^‡^ | KM501392^‡^ | KM501184^‡^ |
| *Hyalopterus pruni* |  |  |  |  | GU457791^‡^ |  | EU358930^‡^ |
| *Aphids kurosawai* |  |  |  |  | GQ904106^‡^ |  | EU358918^‡^ |
| *Aphids aurantii* |  |  |  |  | GU457790^‡^ |  | EU358940^‡^ |
| *Brevicoryne brassicae* |  |  |  |  | GU457798^‡^ |  | EU358928^‡^ |
| *Lipaphis pseudobrassicae* |  |  |  |  | GU457801^‡^ |  | EU358931^‡^ |
| *Eriosoma lanigerum* |  |  |  |  | KP722582^‡^ | KP722582^‡^ | JX559489^‡^ |
| *Cinara costata* |  |  |  |  | JQ916629^‡^ | JX035433^‡^ | KF856819^‡^ |
| *Phylloxerina salicis* |  |  |  |  | JQ920928^‡^ | JX035720^‡^ | JX627598^‡^ |
| *Pineus armandicola* |  |  |  |  | JQ920909^‡^ |  | JX627599^‡^ |
| *Aiceona himalaica* |  |  |  |  | KF856750^‡^ | KP722590^‡^ | KF856821^‡^ |
| *Rhopalosiphum padi* |  |  |  |  | GU457795^‡^ |  | EU358936^‡^ |

^†^Ingroups and outgroups refer to the ingroup and outgroup taxa used in ML analysis.

^‡^Sequences downloaded from GenBank.

**Supplementary Table 3** Grouping information for the Greenideinae samples used in the present study.

| Aphid genus (9 groups) | Number of samples | Voucher |
| --- | --- | --- |
| *Allotrichosiphum* | 2 | 23881, 14135 |
| *Anomalosiphum* | 4 | 21883, 20371, 27195, 20895 |
| *Cervaphis* | 5 | 20216, 19719, 19662, 17351, 24056 |
| *Eutrichosiphum* | 49 | 37277, 36071, BG-Aphid-61, 18534, 17119, 26881, 18623, 18520, 18527, 18459, 39199, 26696, 29567, 32896,  35985, 25670, 34507, 39204, 30436, 14498, 22086, 18609, 21999, 27275, 26278, 19583, 18532, 32525, 21841,  39153, 39245, 39247, 35802, 23753, 17327, 30248, 18549, 37137, 35879, 18485, 20520, 26893, 26262, 39206,  26820, 34616, 37486, 17155, 26852 |
| *Greenidea* | 76 | 14375, 14499, 16276, 17015, 17022, 17527, 18164, 18211, 18497, 18536, 18561, 18593, 18599, 18764, 19024,  19164, 19241, 19373, 19671, 20217, 20528, 20768, 20796, 20824, 20915, 20916, 21738, 21759, 22004, 22132,  22299, 23127, 23718, 23805, 23840, 23931, 23951, 24885, 25623, 25900, 26212, 26223, 26225, 26617, 26656,  26692, 26763, 26772, 26774, 26779, 26837, 26858, 26923, 26931, 27239, 27272, 27449, 27458, 29032, 29035,  29043, 30128, 30153, 30207, 33656, 37145, 38152, 38566, 39055, 39065, 39084, 39119, 39135, 39139,  BG-Aphid-29, BG-Aphid-51 |
| *Greenideoida* | 2 | 26622, 19642 |
| *Mesotrichosiphum* | 1 | 18519 |
| *Mollitrichosiphum* | 65 | 18104, 21910, 21996, 26924, 18324, 23754, 25807, 30244, 30299, 34526, 37222, 37341, 14712, 18382, 23101,  23104, 24859, 30254, 30467, 32700, 35978, 37380, 14560, 14805, 17387, 18502, 18510, 22101, 25613, 26275,  26742, 38180, 38251, 39156, 39213, 39281, BG-aphid-55, 14405, 14417, 18913, 19258, 21845, 21856, 18508,  18513, 20811, 20858, 13361, 14421, 14537, 15381, 18614, 18892, 19242, 19521, 20530, 20938, 22152, 22155,  24067, 26029, 26270, 26906, 26510, 26515 |
| *Schoutedenia* | 11 | 16327, 18056, 22153, 19259, 18927, 26177, 26272, 24121, 24248, 37148, 23912 |
| Aphid species (53 groups) |  |  |
| *Allotrichosiphum cyclobalanopsidis* (Alcyc) | 2 | 14135, 23881 |
| *Anomalosiphum takahashii* (Antak) | 2 | 20371, 21883 |
| *Anomalosiphum tiomanensis* (Antio) | 2 | 27195, 20895 |
| *Cervaphis quercus* (Ceque) | 4 | 20216, 19662, 19719, 17351 |
| *Cervaphis rappardi* (Cerap) | 1 | 24056 |
| *Eutrichosiphum alnicoia* (Eualc) | 1 | 37277 |
| *Eutrichosiphum alnifoliae* (Eualf) | 1 | 36071 |
| *Eutrichosiphum apicifuscum* (Euapi) | 1 | BG-Aphid-61 |
| *Eutrichosiphum dubium* (Eudub) | 1 | 17119 |
| *Eutrichosiphum heterotrichum* (Euhet) | 7 | 18520, 18459, 18527, 18623, 39199, 26696, 26881 |
| *Eutrichosiphum khasyanum* (Eukha) | 3 | 35985, 29567, 32896 |
| *Eutrichosiphum kumaoni* (Eukum) | 2 | 34507, 25670 |
| *Eutrichosiphum parvulum* (Eupar) | 2 | 30436, 39204 |
| *Eutrichosiphum pasaniae* (Eupas) | 16 | 14498, 17327, 18532, 18609, 19583, 21841, 21999, 22086, 23753, 26278, 27275, 32525, 35802, 39153, 39245,  39247 |
| *Eutrichosiphum pseudopasaniae* (Eupse) | 4 | 18549, 30248, 35879, 37137 |
| *Eutrichosiphum sinense* (Eusin) | 6 | 18485, 20520, 26262, 26820, 26893, 39206 |
| *Eutrichosiphum* sp. 1 (Eusp1) | 1 | 34616 |
| *Eutrichosiphum* sp. 2 (Eusp2) | 1 | 18534 |
| *Eutrichosiphum tattakanum* (Eutat) | 3 | 17155, 26852, 37486 |
| *Greenidea anonae* (Grano) | 2 | 20796, 20528 |
| *Greenidea ayyari* (Grayy) | 1 | 18164 |
| *Greenidea brideliae* (Grbri) | 1 | BG-Aphid-29 |
| *Greenidea bucktonis* (Grbuc) | 2 | 23805, 29043 |
| *Greenidea camelliae* (Grcam) | 1 | 26763 |
| *Greenidea castanopsidis* (Grcas) | 4 | 39055, 39119, 37145, 21738 |
| *Greenidea cayratiae* (Grcay) | 7 | 14499, 22004, 25900, 26923, 27239, 27272, 33656 |
| *Greenidea decaspermi* (Grdec) | 2 | 19241, 20915 |
| *Greenidea ficicola* (Grfic) | 17 | 17015, 17022, 18561, 18764, 19671, 20916, 22132, 23718, 23931, 23951, 26212, 27458, 29032, 29035, 30153,  30207, 39065 |
| *Greenidea flacourtiae* (Grfla) | 1 | 18599 |
| *Greenidea kuwanai* (Grkuw) | 16 | 14375, 16276, 17527, 18211, 19024, 19164, 19373, 20217, 21759, 23127, 25623, 26772, 26858, 38152, 38566,  39084 |
| *Greenidea nigra* (Grnig) | 3 | 26837, BG-Aphid-51, 26931 |
| *Greenidea nipponica* (Grnip) | 1 | 24885 |
| *Greenidea prunicola* (Grpru) | 3 | 18593, 18536, 26692 |
| *Greenidea psidii* (Grpsi) | 7 | 20768, 20824, 22299, 26617, 26656, 27449, 30128 |
| *Greenidea querciphaga* (Grque) | 1 | 26223 |
| *Greenidea* sp. 1 (Grsp1) | 1 | 23840 |
| *Greenidea* sp. 2 (Grsp2) | 2 | 26779, 26774 |
| *Greenidea* sp. 3 (Grsp3) | 1 | 26225 |
| *Greenidea* sp. 4 (Grsp4) | 1 | 18497 |
| *Greenidea symplocosis* (Grsym) | 2 | 39139, 39135 |
| *Greenideoida longirostrum* (Grolon) | 1 | 26622 |
| *Greenideoida lutea* (Grolut) | 1 | 19642 |
| *Mesotrichosiphum pentaiarticulatum* (Mepen) | 1 | 18519 |
| *Mollitrichosiphum luchuanum* (Moluc) | 4 | 18104, 21910, 21996, 26924 |
| *Mollitrichosiphum montanum* (Momon) | 8 | 18324, 23754, 25807, 30244, 30299, 34526, 37222, 37341 |
| *Mollitrichosiphum nandii* (Monan) | 10 | 14712, 18382, 23101, 23104, 24859, 30254, 30467, 32700, 35978, 37380 |
| *Mollitrichosiphum nigrofasciatum* (Monig) | 15 | 14560, 14805, 17387, 18502, 18510, 22101, 25613, 26275, 26742, 38180, 38251, 39156, 39213, 39281,  BG-aphid-55 |
| *Mollitrichosiphum nigrum* (Monim) | 6 | 14405, 14417, 18913, 19258, 21845, 21856 |
| *Mollitrichosiphum rhusae* (Morhu) | 4 | 18508, 18513, 20811, 20858 |
| *Mollitrichosiphum tenuicorpus* (Moten) | 16 | 13361, 14421, 14537, 15381, 18614, 18892, 19242, 19521, 20530, 20938, 22152, 22155, 24067, 26029, 26270,  26906 |
| *Mollitrichosiphum tumorisiphum* (Motum) | 2 | 26510, 26515 |
| *Schoutedenia emblica* (Scemb) | 1 | 16327 |
| *Schoutedenia ralumensis* (Scral) | 10 | 24121, 24248, 19259, 18927, 37148, 18056, 22153, 23912, 26177, 26272 |
| Geographic region (32 groups) |  |  |
| Anhui (AH) | 2 | 20216, 20217 |
| Beijing (BJ) | 1 | 19164 |
| Fujian1 (FJ1) | 19 | 26881, 22086, 14498, 26893, 26923, 14499, 22132, 26931, 18104, 26924, 14805, 14560, 22101, 14417, 14405,  14537, 14421, 26906, 18056 |
| Fujian2 (FJ2) | 3 | 22152, 22155, 22153 |
| Guangdong1 (GD1) | 1 | 30153 |
| Guangdong2 (GD2) | 11 | 21883, 18623, 18609, 21999, 22004, 18599, 18593, 21910, 21996, 21856, 18614 |
| Guangdong3 (GD3) | 3 | 19241, 30207, 18561 |
| Guangxi1 (GX1) | 11 | 25670, 27275, 27239, 27272, 19024, 19258, 18913, 26029, 18892, 19259, 18927 |
| Guangxi2 (GX2) | 5 | 26278, 26262, 26275, 26270, 26272 |
| Guangxi3 (GX3) | 1 | 30128 |
| Guangxi4 (GX4) | 6 | 27195, BG-Aphid-61, 26212, 26225, 26223, 26177 |
| Guizhou (GZ) | 5 | 19719, 33656, 16276, 25623, 25613 |
| Hainan (HI) | 38 | 20895, 19662, 18534, 18520, 18459, 18527, 18532, 19583, 32525, 18549, 18485, 20796, 20915, 27458, 19671,  20916, 26622, 19642, 18536, 20768, 20824, 22299, 26617, 26656, 27449, 18497, 18519, 18502, 18510, 18508,  18513, 20811, 20858, 20938, 19521, 19242, 24121, 24248 |
| Hunan1 (HN1) | 1 | 36071 |
| Hunan2 (HN2) | 3 | 21841, 21759, 21845 |
| Hunan3 (HN3) | 1 | 21738 |
| Jiangxi (JX) | 3 | 37137, 37145, 37148 |
| Liaoning (LN) | 2 | 17527, 14375 |
| Nepal (NEP) | 4 | 30248, 30244, 30299, 30254 |
| Sichuan1 (SC1) | 7 | 17119, 17155, 17022, 17015, 23127, 23101, 23104 |
| Sichuan2 (SC2) | 1 | 38566 |
| Shaanxi (SX) | 2 | 37486, 19373 |
| Tibet1 (TB1) | 11 | 35985, 29567, 32896, 35802, 35879, 37341, 18324, 35978, 37380, 18382, 15381 |
| Tibet2 (TB2) | 2 | 37277, 37222 |
| Tibet3 (TB3) | 3 | 25900, 25807, 32700 |
| Taiwan (TW) | 21 | 14135, 39199, 39204, 39245, 39153, 39247, 39206, BG-Aphid-29, 39055, 39119, 39065, 39084, BG-Aphid-51,  39139, 39135, 39156, 39213, 39281, BG-Aphid-55, 26510, 26515 |
| Vietnam (VNM) | 3 | 29043, 29032, 29035 |
| Yunnan1 (YN1) | 9 | 24056, 34507, 34616, 18764, 34526, 14712, 24067, 13361, 16327 |
| Yunnan2 (YN2) | 18 | 23881, 30436, 23753, 20520, 20528, 18164, 23805, 23931, 23718, 23951, 18211, 24885, 23840, 23754, 24859,  30467, 20530, 23912 |
| Zhejiang1 (ZJ1) | 8 | 26696, 26763, 26772, 38152, 26692, 26779, 26774, 26742 |
| Zhejiang2 (ZJ2) | 1 | 38180 |
| Zhejiang3 (ZJ3) | 9 | 20371, 17351, 17327, 26820, 26852, 26858, 26837, 38251, 17387 |
| Host plant (29 groups) |  |  |
| Annonaceae | 1 | 27458 |
| Apocynaceae | 2 | 30436, 39204 |
| Aquifoliaceae | 1 | 14498 |
| Betulaceae | 11 | 37277, 36071, 34526, 37222, 37341, 23754, 14712, 24859, 35978, 37380, 15381 |
| Buxaceae | 1 | 37145 |
| Elaeagnaceae | 1 | 14417 |
| Euphorbiaceae | 11 | BG-Aphid-29, 19259, 27195, 20796, 39055, 39119, 24121, 24248, 18927, 37148, 18056 |
| Fabaceae | 2 | 20371, 21883 |
| Fagaceae | 102 | 14135, 23881, 20216, 19662, 19719, 17351, BG-Aphid-61, 18534, 17119, 18520, 18459, 18527, 18623, 39199,  35985, 29567, 32896, 34507, 25670, 22086, 18532, 19583, 18609, 26278, 39245, 39153, 39247, 32525, 17327,  23753, 35802, 18549, 37137, 35879, 26820, 26893, 20520, 18485, 26262, 39206, 34616, 37486, 17155, 26852,  20528, 18164, 18599, 19373, 16276, 23127, 25623, 38566, 26772, 38152, 17527, 19164, 20217, 39084, 14375,  19024, 26858, 21759, 26837, BG-Aphid-51, 26931, 24885, 18593, 18536, 26692, 18519, 25807, 18382, 18502,  14805, 26742, 38251, 39156, 39213, 39281, BG-Aphid-55, 38180, 18510, 14560, 22101, 17387, 14405, 18513,  20811, 24067, 18614, 26029, 20938, 20530, 14537, 19521, 14421, 18892, 19242, 26270, 26906, 26510, 26515 |
| Juglandaceae | 1 | 39139 |
| Lauraceae | 3 | 26622, 19642, 23840 |
| Lythraceae | 1 | 23805 |
| Meliaceae | 1 | 20858 |
| Moraceae | 16 | 29032, 30207, 39065, 19671, 20916, 23931, 18764, 22132, 23718, 23951, 26212, 30153, 17022, 18561, 29035,  26779 |
| Myricaceae | 1 | 39135 |
| Myrtaceae | 8 | 29043, 20768, 20824, 22299, 26617, 26656, 27449, 30128 |
| Oleaceae | 1 | 17015 |
| Proteaceae | 1 | 18508 |
| Rhamnaceae | 1 | 26774 |
| Rosaceae | 1 | 18211 |
| Rutaceae | 1 | 21738 |
| Sabiaceae | 3 | 18104, 18913, 13361 |
| Sapindaceae | 3 | 27272, 19241, 20915 |
| Sapotaceae | 1 | 18497 |
| Simaroubaceae | 1 | 19258 |
| Theaceae | 1 | 26763 |
| Thelypteridaceae | 1 | 16327 |
| Verbenaceae | 1 | 21999 |
| Vitaceae | 4 | 26923, 27239, 25900, 14499 |

**Supplementary Table 4** Relative abundance of the top ten bacterial phyla, classes, orders, families and genera in Greenideinae.

| Phylum | Class | Order | Family | Genus |
| --- | --- | --- | --- | --- |
| Proteobacteria/98.74% | Gammaproteobacteria/95.15% | Enterobacteriales/94.72% | Enterobacteriaceae/94.42% | ***Buchnera***/68.19% |
| Bacteroidetes/0.92% | Alphaproteobacteria/3.56% | Rickettsiales/3.56% | Anaplasmataceae/2.10% | ***Serratia***/14.97% |
| Firmicutes/0.28% | Flavobacteriia/0.88% | Flavobacteriales/0.88% | Rickettsiaceae/1.34% | ***Wolbachia***/2.10% |
| Actinobacteria/0.02% | Clostridia/0.14% | Clostridiales/0.13% | Blattabacteriaceae/0.88% | ***Arsenophonus***/1.80% |
| Deinococcus–Thermus/0.02% | Bacilli/0.12% | Bacillales/0.09% | Yersiniaceae/0.30% | ***Rickettsia***/1.34% |
| Fusobacteria/0.01% | Bacteroidia/0.04% | Orbales/0.07% | Bacillaceae/0.09% | *Pectobacterium*/0.44% |
|  | Erysipelotrichia/0.03% | Pseudomonadales/0.05% | Orbaceae/0.07% | ***Hamiltonella***/0.41% |
|  | Deinococci/0.02% | Bacteroidales/0.04% | Lachnospiraceae/0.07% | ***Fukatsuia***/0.30% |
|  | Fusobacteriia/0.01% | Erysipelotrichales/0.03% | Moraxellaceae/0.04% | *Escherichia–Shigella*/0.15% |
|  | unidentified_Actinobacteria/0.01% | Lactobacillales/0.02% | Ruminococcaceae/0.04% | *Pantoea*/0.05% |

Aphid symbionts are indicated in bold.

**Supplementary Table 5** Infection pattern of secondary symbionts within Greenideinae.

| Secondary symbiont | Infection frequency | Aphid species |
| --- | --- | --- |
| *Serratia-Wolbachia* | 9/215 | *Eutrichosiphum apicifuscum*, *Eutrichosiphum heterotrichum*, *Eutrichosiphum pseudopasaniae*, *Greenidea kuwanai*,  *Greenidea nigra*, *Greenidea prunicola*, *Schoutedenia ralumensis* |
| *Serratia-Wolbachia-Arsenophonus* | 50/215 | *Allotrichosiphum cyclobalanopsidis*, *Anomalosiphumi takahashii*, *Anomalosiphum tiomanensis*, *Cervaphis quercus*,  *Cervaphis rappardi*, *Eutrichosiphum heterotrichum*, *Eutrichosiphum kumaoni*, *Eutrichosiphum pasaniae*,  *Eutrichosiphum sinense*, *Eutrichosiphum tattakanum*, *Greenidea anonae*, *Greenidea brideliae*, *Greenidea bucktonis*,  *Greenidea castanopsidis*, *Greenidea cayratiae*, *Greenidea decaspermi*, *Greenidea ficicola*, *Greenidea kuwanai*,  *Greenidea nigra*, *Greenidea nipponica*, *Greenidea psidii*, *Greenidea* sp. 1, *Greenidea* sp. 2, *Schoutedenia ralumensis* |
| *Serratia-Wolbachia-Fukatsuia* | 1/215 | *Eutrichosiphum pasaniae* |
| *Serratia-Wolbachia-Hamiltonella* | 3/215 | *Greenidea* sp. 1, *Schoutedenia ralumensis* |
| *Serratia-Wolbachia-Rickettsia* | 14/215 | *Cervaphis quercus*, *Eutrichosiphum heterotrichum*, *Eutrichosiphum pasaniae*, *Eutrichosiphum sinense*,  *Greenidea ayyari*, *Greenidea decaspermi*, *Greenidea ficicola*, *Greenidea kuwanai*,  *Mesotrichosiphum pentaiarticulatum*, *Schoutedenia ralumensis* |
| *Serratia-Wolbachia-Arsenophonus-Fukatsuia* | 12/215 | *Eutrichosiphum pasaniae*, *Eutrichosiphum sinense*, *Greenidea camelliae*, *Greenidea cayratiae*, *Greenidea ficicola*,  *Greenidea* sp. 3, *Greenideoida longirostrum*, *Greenidea psidii*, *Greenidea querciphaga*,  *Schoutedenia ralumensis* |
| *Serratia-Wolbachia-Arsenophonus-Hamiltonella* | 18/215 | *Eutrichosiphum alnifoliae*, *Eutrichosiphum heterotrichum*, *Eutrichosiphum khasyanum*, *Eutrichosiphum kumaoni*,  *Eutrichosiphum parvulum*, *Eutrichosiphum pasaniae*, *Eutrichosiphum pseudopasaniae*, *Greenidea bucktonis*,  *Greenidea camelliae*, *Greenidea castanopsidis*, *Greenidea ficicola*, *Greenidea* sp. 3, *Mollitrichosiphum nigrum*,  *Mollitrichosiphum rhusae*, *Mollitrichosiphum tenuicorpus*, *Schoutedenia ralumensis* |
| *Serratia-Wolbachia-Arsenophonus-Rickettsia* | 11/215 | *Cervaphis quercus*, *Greenidea ficicola*, *Mollitrichosiphum nigrofasciatum*, *Mollitrichosiphum nigrum*,  *Mollitrichosiphum rhusae*, *Mollitrichosiphum tenuicorpus* |
| *Serratia-Wolbachia-Fukatsuia-Hamiltonella* | 4/215 | *Eutrichosiphum pasaniae*, *Eutrichosiphum tattakanum*, *Greenidea cayratiae*, *Schoutedenia emblica* |
| *Serratia-Wolbachia-Fukatsuia-Rickettsia* | 2/215 | *Greenidea flacourtiae*, *Greenidea* sp. 4 |
| *Serratia-Wolbachia-Rickettsia-Hamiltonella* | 6/215 | *Eutrichosiphum* sp. 2, *Eutrichosiphum heterotrichum*, *Eutrichosiphum pasaniae*, *Greenidea kuwanai*,  *Greenideoida lutea* |
| *Serratia-Wolbachia-Arsenophonus-Fukatsuia-Hamiltonella* | 22/215 | *Eutrichosiphum alnicoia*, *Eutrichosiphum khasyanum*, *Eutrichosiphum pasaniae*,  *Eutrichosiphum pseudopasaniae*, *Eutrichosiphum sinense*, *Eutrichosiphum* sp. 1, *Eutrichosiphum tattakanum*,  *Greenidea castanopsidis*, *Greenidea cayratiae*, *Greenidea ficicola*, *Greenidea kuwanai*, *Greenidea psidii*,  *Greenidea symplocosis*, *Schoutedenia ralumensis* |
| *Serratia-Wolbachia-Arsenophonus-Fukatsuia-Rickettsia* | 28/215 | *Mollitrichosiphum luchuanum*, *Mollitrichosiphum montanum*, *Mollitrichosiphum nandii*,  *Mollitrichosiphum nigrofasciatum*, *Mollitrichosiphum tenuicorpus* |
| *Serratia-Wolbachia-Arsenophonus-Hamiltonella-Rickettsia* | 18/215 | *Greenidea kuwanai*, *Greenidea nigra*, *Mollitrichosiphum nigrofasciatum*, *Mollitrichosiphum nigrum*,  *Mollitrichosiphum rhusae*, *Mollitrichosiphum tenuicorpus*, *Mollitrichosiphum tumorisiphum* |
| *Serratia-Wolbachia-Fukatsuia-Hamiltonella-Rickettsia* | 5/215 | *Allotrichosiphum cyclobalanopsidis*, *Eutrichosiphum dubium*, *Greenidea ficicola*, *Greenidea kuwanai* |
| *Serratia-Wolbachia-Arsenophonus-Fukatsuia-Hamiltonella-Rickettsia* | 12/215 | *Mollitrichosiphum luchuanum*, *Mollitrichosiphum montanum*, *Mollitrichosiphum nandii*,  *Mollitrichosiphum nigrofasciatum, Mollitrichosiphum rhusae, Mollitrichosiphum tenuicorpus,*  *Mollitrichosiphum tumorisiphum* |

**Supplementary Table 6** Relative contribution of aphid genus, geography and host plant on microbial communities estimated by three-way ANOVA.

| Alpha diversity index | Sample size | Source of variation | Df | Bacteria | | | |  | Symbionts | | | |  | Secondary symbionts | | | |
| --- | --- | --- | --- | --- | --- | --- | --- | --- | --- | --- | --- | --- | --- | --- | --- | --- | --- |
|  |  |  |  | SS | MS | *F* | *P* |  | SS | MS | *F* | *P* |  | SS | MS | *F* | *P* |
| Shannon | n ≥ 2 | Aphid genus | 7 | 0.718 | 0.103 | 4.292 | *<0.001* |  | 0.573 | 0.082 | 3.727 | *<0.001* |  | 0.855 | 0.122 | 2.068 | *0.049* |
|  |  | Geographic region | 21 | 0.764 | 0.036 | 1.500 | 0.104 |  | 0.707 | 0.034 | 1.545 | 0.109 |  | 0.922 | 0.044 | 0.746 | 0.893 |
|  |  | Aphid genus: Geographic region | 31 | 1.343 | 0.043 | 1.792 | *0.006* |  | 1.153 | 0.037 | 1.682 | *0.029* |  | 2.232 | 0.072 | 1.220 | 0.289 |
|  |  | Host plant | 10 | 0.391 | 0.033 | 1.375 | 0.127 |  | 0.330 | 0.028 | 1.273 | 0.197 |  | 0.646 | 0.054 | 0.915 | 0.636 |
|  |  | Geographic region: Host plant | 14 | 0.192 | 0.014 | 0.583 | 1.000 |  | 0.222 | 0.016 | 0.727 | 0.724 |  | 1.002 | 0.072 | 1.220 | 0.357 |
|  |  | Residual | 70 | 1.653 | 0.024 |  |  |  | 1.552 | 0.022 |  |  |  | 4.151 | 0.059 |  |  |
|  | n ≥ 3 | Aphid genus | 4 | 0.750 | 0.188 | 7.833 | *<0.001* |  | 0.571 | 0.143 | 6.212 | *<0.001* |  | 0.842 | 0.210 | 3.559 | *0.014* |
|  |  | Geographic region | 14 | 0.668 | 0.048 | 2.000 | *0.006* |  | 0.622 | 0.044 | 1.913 | *0.005* |  | 0.547 | 0.039 | 0.661 | 0.865 |
|  |  | Aphid genus: Geographic region | 22 | 0.977 | 0.044 | 1.833 | *0.026* |  | 0.839 | 0.038 | 1.652 | 0.051 |  | 2.048 | 0.093 | 1.576 | 0.051 |
|  |  | Host plant | 8 | 0.109 | 0.014 | 0.583 | 0.546 |  | 0.101 | 0.013 | 0.565 | 0.439 |  | 0.405 | 0.051 | 0.864 | 0.552 |
|  |  | Geographic region: Host plant | 13 | 0.192 | 0.015 | 0.625 | 0.851 |  | 0.221 | 0.017 | 0.739 | 0.681 |  | 1.002 | 0.077 | 1.305 | 0.171 |
|  |  | Residual | 69 | 1.643 | 0.024 |  |  |  | 1.555 | 0.023 |  |  |  | 4.056 | 0.059 |  |  |
| Simpson | n ≥ 2 | Aphid genus | 7 | 0.334 | 0.048 | 2.667 | *0.013* |  | 0.300 | 0.043 | 2.048 | 0.056 |  | 0.611 | 0.087 | 1.891 | 0.052 |
|  |  | Geographic region | 21 | 0.495 | 0.024 | 1.333 | 0.272 |  | 0.586 | 0.028 | 1.333 | 0.215 |  | 0.753 | 0.036 | 0.783 | 0.863 |
|  |  | Aphid genus: Geographic region | 31 | 1.000 | 0.032 | 1.778 | *0.014* |  | 0.998 | 0.032 | 1.524 | 0.051 |  | 1.658 | 0.053 | 1.152 | 0.350 |
|  |  | Host plant | 10 | 0.241 | 0.020 | 1.111 | 0.213 |  | 0.218 | 0.018 | 0.857 | 0.474 |  | 0.529 | 0.044 | 0.957 | 0.593 |
|  |  | Geographic region: Host plant | 14 | 0.141 | 0.010 | 0.556 | 1.000 |  | 0.171 | 0.012 | 0.571 | 0.817 |  | 0.775 | 0.055 | 1.196 | 0.315 |
|  |  | Residual | 70 | 1.253 | 0.018 |  |  |  | 1.447 | 0.021 |  |  |  | 3.230 | 0.046 |  |  |
|  | n ≥ 3 | Aphid genus | 4 | 0.382 | 0.096 | 5.333 | *<0.001* |  | 0.333 | 0.083 | 3.952 | *0.002* |  | 0.631 | 0.158 | 3.435 | *0.014* |
|  |  | Geographic region | 14 | 0.499 | 0.036 | 2.000 | *0.019* |  | 0.533 | 0.038 | 1.810 | *0.027* |  | 0.461 | 0.033 | 0.717 | 0.851 |
|  |  | Aphid genus: Geographic region | 22 | 0.824 | 0.037 | 2.056 | *0.029* |  | 0.795 | 0.036 | 1.714 | 0.070 |  | 1.564 | 0.071 | 1.543 | 0.055 |
|  |  | Host plant | 8 | 0.092 | 0.011 | 0.611 | 0.553 |  | 0.082 | 0.010 | 0.476 | 0.526 |  | 0.354 | 0.044 | 0.957 | 0.555 |
|  |  | Geographic region: Host plant | 13 | 0.141 | 0.011 | 0.611 | 0.748 |  | 0.171 | 0.013 | 0.619 | 0.775 |  | 0.774 | 0.060 | 1.304 | 0.211 |
|  |  | Residual | 69 | 1.233 | 0.018 |  |  |  | 1.436 | 0.021 |  |  |  | 3.200 | 0.046 |  |  |

Statistically significant *P* values (*P* < 0.05) are highlighted in italics. Abbreviations: Df, degree of freedom; SS, sum of squares; MS, mean sum of squares; ‘:’, interaction. The effects of ‘Aphid genus: Host plant’ and ‘Aphid genus: Geographic region: Host plant’ were not detected.

**Supplementary Table 7** Relative contribution of aphid species, geography and host plant on microbial communities estimated by three-way ANOVA.

| Alpha diversity index | Sample size | Source of variation | Df | Bacteria | | | |  | Symbionts | | | |  | Secondary symbionts | | | | |
| --- | --- | --- | --- | --- | --- | --- | --- | --- | --- | --- | --- | --- | --- | --- | --- | --- | --- | --- |
|  |  |  |  | SS | MS | *F* | *P* |  | SS | MS | *F* | *P* |  | SS | MS | *F* | *P* | |
| Shannon | n ≥ 2 | Aphid species | 28 | 2.551 | 0.091 | 11.375 | *<0.001* |  | 2.400 | 0.086 | 12.286 | *<0.001* |  | 2.475 | 0.088 | 1.419 | | 0.174 |
|  |  | Geographic region | 21 | 0.353 | 0.017 | 2.125 | *0.012* |  | 0.229 | 0.011 | 1.571 | 0.063 |  | 0.943 | 0.045 | 0.726 | | 0.851 |
|  |  | Aphid species: Geographic region | 47 | 0.742 | 0.016 | 2.000 | *0.016* |  | 0.674 | 0.014 | 2.000 | *0.012* |  | 2.408 | 0.051 | 0.823 | | 0.825 |
|  |  | Host plant | 5 | 0.012 | 0.002 | 0.250 | 1.000 |  | 0.005 | 0.001 | 0.143 | 1.000 |  | 0.074 | 0.015 | 0.242 | | 0.906 |
|  |  | Residual | 38 | 0.308 | 0.008 |  |  |  | 0.276 | 0.007 |  |  |  | 2.369 | 0.062 |  | |  |
|  | n ≥ 3 | Aphid species | 15 | 2.230 | 0.149 | 18.625 | *<0.001* |  | 2.186 | 0.146 | 18.250 | *<0.001* |  | 1.199 | 0.080 | 1.356 | | 0.232 |
|  |  | Geographic region | 11 | 0.172 | 0.016 | 2.000 | 0.073 |  | 0.139 | 0.013 | 1.625 | 0.133 |  | 0.476 | 0.043 | 0.729 | | 0.895 |
|  |  | Aphid species: Geographic region | 35 | 0.555 | 0.016 | 2.000 | *0.031* |  | 0.520 | 0.015 | 1.875 | *0.033* |  | 1.975 | 0.056 | 0.949 | | 0.601 |
|  |  | Host plant | 1 | 0.001 | 0.001 | 0.125 | 1.000 |  | 0.004 | 0.004 | 0.500 | 0.594 |  | 0.040 | 0.040 | 0.678 | | 0.528 |
|  |  | Residual | 33 | 0.254 | 0.008 |  |  |  | 0.251 | 0.008 |  |  |  | 1.959 | 0.059 |  | |  |
| Simpson | n ≥ 2 | Aphid species | 28 | 1.557 | 0.056 | 9.333 | *<0.001* |  | 1.774 | 0.063 | 10.500 | *<0.001* |  | 1.674 | 0.060 | 1.200 | | 0.308 |
|  |  | Geographic region | 21 | 0.189 | 0.009 | 1.500 | 0.091 |  | 0.169 | 0.008 | 1.333 | 0.120 |  | 0.725 | 0.035 | 0.700 | | 0.832 |
|  |  | Aphid species: Geographic region | 47 | 0.480 | 0.010 | 1.667 | 0.072 |  | 0.537 | 0.011 | 1.833 | *0.028* |  | 1.764 | 0.038 | 0.760 | | 0.924 |
|  |  | Host plant | 5 | 0.017 | 0.003 | 0.500 | 0.873 |  | 0.002 | 0.0004 | 0.067 | 1.000 |  | 0.074 | 0.015 | 0.300 | | 0.921 |
|  |  | Residual | 38 | 0.233 | 0.006 |  |  |  | 0.236 | 0.006 |  |  |  | 1.884 | 0.050 |  | |  |
|  | n ≥ 3 | Aphid species | 15 | 1.465 | 0.098 | 19.600 | *<0.001* |  | 1.618 | 0.108 | 18.000 | *<0.001* |  | 0.931 | 0.062 | 1.292 | | 0.244 |
|  |  | Geographic region | 11 | 0.105 | 0.010 | 2.000 | 0.125 |  | 0.110 | 0.010 | 1.667 | 0.134 |  | 0.366 | 0.033 | 0.688 | | 0.817 |
|  |  | Aphid species: Geographic region | 35 | 0.404 | 0.012 | 2.400 | *0.033* |  | 0.449 | 0.013 | 2.167 | *0.023* |  | 1.366 | 0.039 | 0.813 | | 0.717 |
|  |  | Host plant | 1 | 0.0008 | 0.0008 | 0.160 | 1.000 |  | 0.001 | 0.001 | 0.167 | 0.594 |  | 0.053 | 0.053 | 1.104 | | 0.528 |
|  |  | Residual | 33 | 0.177 | 0.005 |  |  |  | 0.198 | 0.006 |  |  |  | 1.599 | 0.048 |  | |  |

Statistically significant *P* values (*P* < 0.05) are highlighted in italics. Abbreviations: Df, degree of freedom; SS, sum of squares; MS, mean sum of squares; ‘:’, interaction. The effects of ‘Aphid species: Host plant’, ‘Geographic region: Host plant’ and ‘Aphid species: Geographic region: Host plant’ were not detected.

**Supplementary Table 8** Mean posterior probabilities (PPs) for aphid-secondary symbiont association states estimated in Bayesian reconstruction.

| Node | *Serratia symbiotica* | *Wolbachia* | *Arsenophonus* | *Rickettsia* | *Hamiltonella defensa* | *Fukatsuia symbiotica* |
| --- | --- | --- | --- | --- | --- | --- |
| 1 | **0.474879149** | **0.493772136** | 0.019454436 | 0.004720457 | 0.003751961 | 0.003421827 |
| 2 | **0.479362235** | **0.497660809** | 0.011707547 | 0.007104616 | 0.002201657 | 0.001963112 |
| 3 | **0.484270088** | **0.502298876** | 0.006751956 | 0.002153359 | 0.002370027 | 0.002155677 |
| 4 | **0.333279316** | **0.333109491** | **0.325579019** | 0.002693467 | 0.002821049 | 0.002517641 |
| 5 | **0.490452063** | **0.506791405** | 0.000896035 | 0.000628876 | 0.000692615 | 0.00053902 |
| 6 | **0.493357573** | **0.495690116** | 0.008898716 | 0.000808888 | 0.000864061 | 0.000380657 |
| 7 | **0.322384423** | **0.322313753** | **0.319222203** | 0.016549225 | 0.017001565 | 0.002528827 |
| 8 | **0.197298793** | **0.197303004** | **0.196909847** | **0.196675165** | **0.196765608** | 0.015047589 |
| 9 | **0.497172125** | **0.498866517** | 0.003076725 | 0.000292051 | 0.000307509 | 0.000285072 |
| 10 | **0.499149501** | **0.500602632** | 7.8204E-05 | 6.10973E-05 | 3.78253E-05 | 7.07053E-05 |
| 11 | **0.498616355** | **0.499426901** | 0.001444943 | 8.386E-05 | 0.000205323 | 0.00022252 |
| 12 | **0.479682472** | **0.480056817** | 0.015804316 | 0.004225537 | 0.004593301 | 0.015637552 |
| 13 | **0.479310455** | **0.479450237** | 0.012451395 | 0.015898081 | 0.000672095 | 0.012217721 |
| 14 | **0.485687425** | **0.485897443** | 0.022047897 | 0.000298969 | 0.005648783 | 0.000419511 |
| 15 | **0.488360027** | **0.488405977** | 0.019143881 | 0.000116637 | 0.002005029 | 0.001968409 |
| 16 | **0.491218453** | **0.491176001** | 0.017577412 | 0.000001188 | 8.28E-07 | 0.000026028 |
| 17 | **0.327219595** | **0.327049139** | **0.32268752** | 0.000224971 | 0.000233387 | 0.022585413 |
| 18 | **0.250280608** | **0.250228764** | **0.249358172** | 0.000224736 | 0.000230357 | **0.249677359** |
| 19 | **0.490574379** | **0.49070148** | 0.018315005 | 0.000143203 | 9.61E-05 | 0.000169837 |
| 20 | **0.485833644** | **0.485998112** | 0.017578837 | 0.003834645 | 0.002030291 | 0.00472446 |
| 21 | **0.487030452** | **0.487176588** | 0.017056389 | 0.003225087 | 0.001390132 | 0.004121341 |
| 22 | **0.329103497** | **0.329058312** | **0.325908531** | 0.000219901 | 0.014797681 | 0.000912088 |
| 23 | **0.248308061** | **0.248320415** | **0.247083147** | 0.000664548 | **0.246940052** | 0.008683772 |
| 24 | **0.320865035** | **0.320814257** | **0.319630179** | 0.011346095 | 0.014100852 | 0.013243544 |
| 25 | **0.491646869** | **0.49193792** | 0.000285712 | 0.007903969 | 0.00030086 | 0.007924703 |
| 26 | **0.488373899** | **0.500705901** | 0.001408772 | 0.003864643 | 0.003902139 | 0.001744652 |
| 27 | **0.391601585** | **0.395305715** | 0.017923115 | 0.1129905 | 0.041448203 | 0.040730861 |
| 28 | **0.231643475** | **0.232401537** | 0.037369821 | 0.038531412 | **0.229177741** | **0.230875999** |
| 29 | **0.487423739** | **0.494976968** | 0.003372104 | 0.001352571 | 0.011576675 | 0.001297905 |
| 30 | **0.396274561** | **0.397299253** | 0.04918534 | 0.054058587 | 0.054760801 | 0.048421468 |
| 31 | **0.473681291** | **0.478453888** | 0.008078344 | 0.000535392 | 0.03868756 | 0.000563553 |
| 32 | **0.477805329** | **0.480918217** | 0.00211192 | 0.001639295 | 0.036414215 | 0.001111037 |
| 33 | **0.43941326** | **0.440353591** | 0.02988596 | 0.029812268 | 0.030604848 | 0.029930055 |
| 34 | **0.332703856** | **0.334027561** | 0.006896876 | 0.004190592 | **0.320349203** | 0.001831899 |
| 35 | **0.320330924** | **0.321368689** | 0.004391861 | 0.035196083 | **0.31458128** | 0.004131173 |
| 36 | **0.312493647** | **0.312816617** | 0.020912567 | 0.023406484 | **0.310307576** | 0.020063117 |
| 37 | **0.244664291** | **0.244794244** | 0.010165951 | 0.012043889 | **0.24394622** | **0.244385401** |
| 38 | **0.243164211** | **0.243287161** | **0.239895723** | 0.005524925 | **0.239494411** | 0.028633567 |
| 39 | **0.196254341** | **0.196287775** | **0.195492532** | 0.020757081 | **0.195472676** | **0.195735613** |
| 40 | **0.238480825** | **0.238540528** | **0.236599199** | 0.025636676 | **0.236279036** | 0.024463732 |
| 41 | **0.252261185** | **0.252649785** | **0.242596316** | 0.005022801 | **0.241235961** | 0.00623396 |
| 42 | **0.245936059** | **0.246005591** | **0.242774511** | 0.001318337 | **0.242433568** | 0.021531968 |
| 43 | **0.244121313** | **0.244164903** | **0.241907747** | 0.007936868 | **0.241613969** | 0.020255172 |
| 44 | **0.246900132** | **0.24692802** | **0.24508266** | 0.001403239 | **0.244854983** | 0.014831003 |
| 45 | **0.241775268** | **0.241953383** | **0.237187593** | 0.026068999 | **0.236411457** | 0.016603329 |
| 46 | **0.246555529** | **0.24668766** | **0.242312187** | 0.016395385 | **0.241595096** | 0.006454157 |
| 47 | **0.198360392** | **0.198363507** | **0.198115444** | **0.197967259** | **0.198022943** | 0.009170459 |
| 48 | **0.166667** | **0.166667** | **0.166667** | **0.166667** | **0.166667** | **0.166667** |
| 49 | **0.241318369** | **0.241433167** | **0.238176363** | 0.013245312 | **0.237783671** | 0.028043109 |
| 50 | **0.242775879** | **0.242876179** | **0.239790032** | 0.009839688 | **0.239426131** | 0.025292101 |
| 51 | **0.245389885** | **0.245468891** | **0.242713437** | 0.003681871 | **0.242404912** | 0.020340933 |
| 52 | **0.166667** | **0.166667** | **0.166667** | **0.166667** | **0.166667** | **0.166667** |

Node numbers refer to those in **Figure 6**. Bold numbers indicate PPs of the optimal states. The results of the three runs were significantly similar, and only one of them is reported here.

**Supplementary Figure legends**

**Supplementary Figure 1** Heatmap representing the distribution and relative abundances of *Buchnera* OTUs among Greenideinae aphids. The maximum-likelihood trees display the phylogenetic relationships of *Buchnera* OTUs and Greenideinae.

**Supplementary Figure 2** Heatmap representing the distribution and relative abundances of *Arsenophonus* OTUs among Greenideinae aphids. The maximum-likelihood trees display the phylogenetic relationships of *Arsenophonus* OTUs and Greenideinae.

**Supplementary Figure 3** Heatmap representing the distribution and relative abundances of OTUs belonging to *Rickettsia* **(A)**, *Hamiltonella defensa* **(B)** and *Fukatsuia symbiotica* **(C)**. The maximum-likelihood trees display the phylogenetic relationships of secondary symbiont OTUs and Greenideinae.

**Supplementary Figure 4** Nonmetric multidimensional scaling (NMDS) plots based on Bray–Curtis distances of bacterial **(A, D, G, J)**, symbiont **(B, E, H, K)** and secondary symbiont **(C, F, I, L)** communities (n ≥ 1). Samples are colored by aphid genus **(A–C)**, aphid species **(D–F)**, geographic region **(G–I)** and host plant **(J–L)**. The stress value indicates the goodness of fit between the NMDS representation and the data. The abbreviations are given in **Supplementary Table 3**.

**Supplementary Figure 5** Nonmetric multidimensional scaling (NMDS) plots based on Bray–Curtis distances of bacterial **(A, D, G, J)**, symbiont **(B, E, H, K)** and secondary symbiont **(C, F, I, L)** communities (n ≥ 3). Samples are colored by aphid genus **(A–C)**, aphid species **(D–F)**, geographic region **(G–I)** and host plant **(J–L)**. The stress value indicates the goodness of fit between the NMDS representation and the data. The abbreviations are given in **Supplementary Table 3**.

**Supplementary Figure 6** Nonmetric multidimensional scaling (NMDS) plots based on unweighted UniFrac distances of bacterial **(A, D, G, J)**, symbiont **(B, E, H, K)** and secondary symbiont **(C, F, I, L)** communities (n ≥ 1). Samples are colored by aphid genus **(A–C)**, aphid species **(D–F)**, geographic region **(G–I)** and host plant **(J–L)**. The stress value indicates the goodness of fit between the NMDS representation and the data. The abbreviations are given in **Supplementary Table 3**.

**Supplementary Figure 7** Nonmetric multidimensional scaling (NMDS) plots based on unweighted UniFrac distances of bacterial **(A, D, G, J)**, symbiont **(B, E, H, K)** and secondary symbiont **(C, F, I, L)** communities (n ≥ 3). Samples are colored by aphid genus **(A–C)**, aphid species **(D–F)**, geographic region **(G–I)** and host plant **(J–L)**. The stress value indicates the goodness of fit between the NMDS representation and the data. The abbreviations are given in **Supplementary Table 3**.

**Supplementary Figure 8** Structural segregation using constrained principal coordinate analyses (cPCoA) of Bray–Curtis distances of bacterial **(A, D, G, J)**, symbiont **(B, E, H, K)** and secondary symbiont **(C, F, I, L)** communities (n ≥ 1). Samples are colored by aphid genus **(A–C)**, aphid species **(D–F)**, geographic region **(G–I)** and host plant **(J–L)**. The abbreviations are given in **Supplementary Table 3**.

**Supplementary Figure 9** Ancestral aphid-secondary symbiont associations estimated by parsimony reconstruction. The absence (white) or presence (colors) of secondary symbionts was mapped on the simplified cladogram displaying the phylogenetic relationships of Greenideinae species. Nodes that are not fully coloured refer to uncertain ancestral state.
