# Supplementary material for: Insights Into the Species-Specific Microbiota of Greenideinae (Hemiptera: Aphididae) With Evidence of Phylosymbiosis
Source: Front Microbiol. 2022 Feb 22;13:828170. doi: 10.3389/fmicb.2022.828170 (PMC8901875; doi:10.3389/fmicb.2022.828170)
Supplement: Supplementary file 3 [file Data_Sheet_2.PDF]

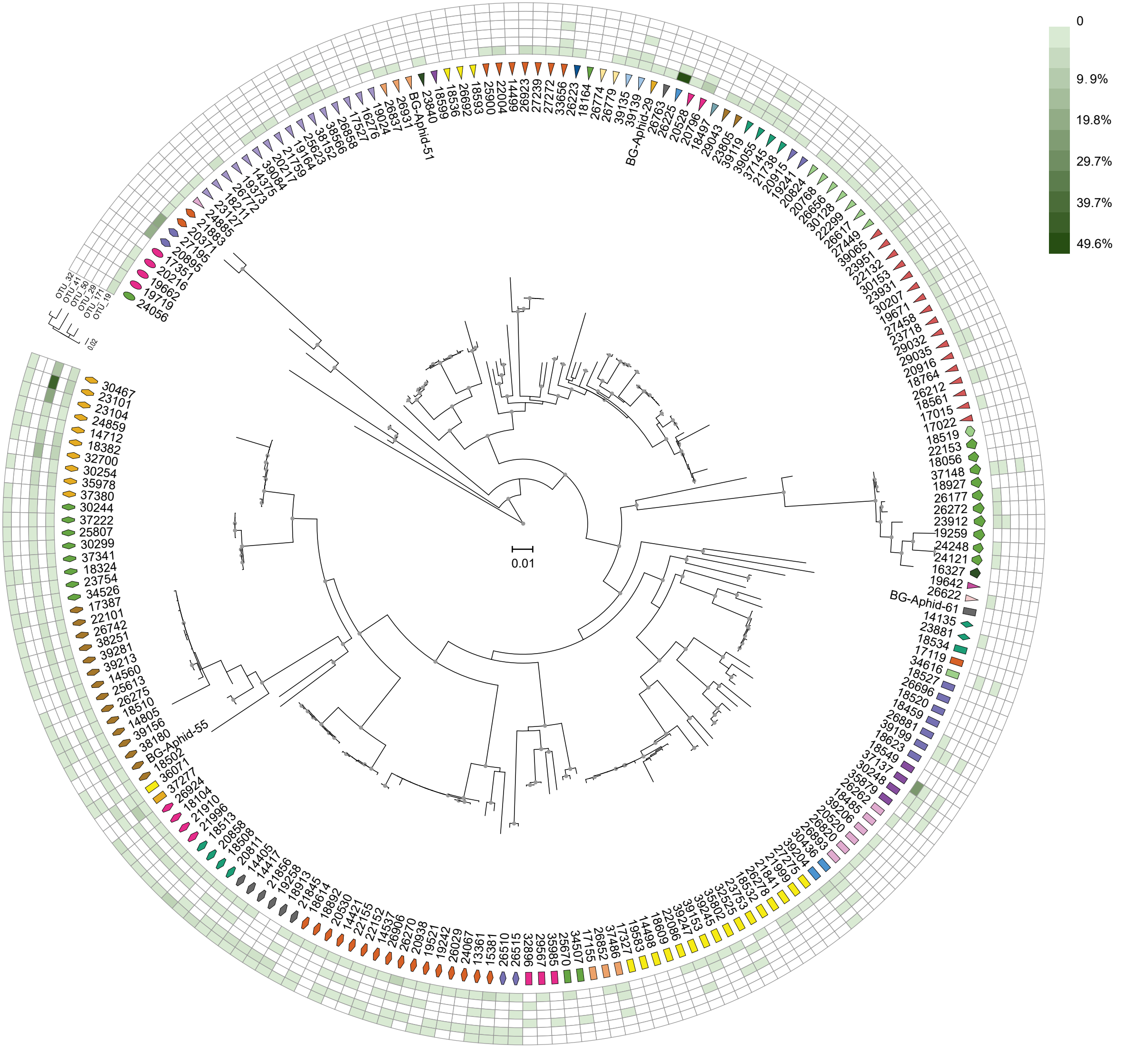

Supplementary Figure 2 Heatmap representing the distribution and relative abundances of *Arsenophonus* OTUs among Greenideinae aphids. The maximum-likelihood trees display the phylogenetic relationships of *Arsenophonus* OTUs and Greenideinae.
